# Molecular Mechanisms Underlying the Enhanced Analgesic Effect of Oxycodone Compared to Morphine in Chemotherapy-Induced Neuropathic Pain

**DOI:** 10.1371/journal.pone.0091297

**Published:** 2014-03-11

**Authors:** Karine Thibault, Bernard Calvino, Isabelle Rivals, Fabien Marchand, Sophie Dubacq, Stephen B. McMahon, Sophie Pezet

**Affiliations:** 1 Brain Plasticity Unit, ESPCI-ParisTech, Paris, France; 2 Centre National de la Recherche Scientifique, UMR 8249, Paris, France; 3 Neurorestoration Group, The Wolfson Centre for Age-Related Diseases, King's College London, London, United Kingdom; 4 Equipe de Statistique Appliquée, ESPCI-ParisTech, Paris, France; 5 Institut National de la Santé et de la Recherche Médicale, Unité 1107, NEURO-DOL, Clermont-Ferrand, France; 6 Clermont Université, Université d'Auvergne, Pharmacologie Fondamentale et Clinique de la Douleur, Clermont-Ferrand, France; Boston Children's Hospital and Harvard Medical School, United States of America

## Abstract

Oxycodone is a μ-opioid receptor agonist, used for the treatment of a large variety of painful disorders. Several studies have reported that oxycodone is a more potent pain reliever than morphine, and that it improves the quality of life of patients. However, the neurobiological mechanisms underlying the therapeutic action of these two opioids are only partially understood. The aim of this study was to define the molecular changes underlying the long-lasting analgesic effects of oxycodone and morphine in an animal model of peripheral neuropathy induced by a chemotherapic agent, vincristine. Using a behavioural approach, we show that oxycodone maintains an optimal analgesic effect after chronic treatment, whereas the effect of morphine dies down. In addition, using DNA microarray technology on dorsal root ganglia, we provide evidence that the long-term analgesic effect of oxycodone is due to an up-regulation in GABA_B_ receptor expression in sensory neurons. These receptors are transported to their central terminals within the dorsal horn, and subsequently reinforce a presynaptic inhibition, since only the long-lasting (and not acute) anti-hyperalgesic effect of oxycodone was abolished by intrathecal administration of a GABA_B_ receptor antagonist; in contrast, the morphine effect was unaffected. Our study demonstrates that the GABA_B_ receptor is functionally required for the alleviating effect of oxycodone in neuropathic pain condition, thus providing new insight into the molecular mechanisms underlying the sustained analgesic action of oxycodone.

## Introduction

Approximately 7–8% of the European population suffers from neuropathic pain, and 5% of these cases may be severe [1]. Unfortunately, the pharmacotherapy of neuropathic pain is frequently unsatisfactory for patients. One reason for their poor efficacy is the lack of a clear understanding of the neurobiological mechanisms that underlie neuropathic pain. Morphine is the most commonly used opioid analgesic in the past 30 years. However, repeated use of opioids induces a tolerance phenomenon, which significantly reduces their analgesic effects. Among clinically used opioids, oxycodone shows excellent anti-hyperalgesic and anti-allodynic effects against neuropathic pain [2], [3]. Oxycodone is a semi-synthetic opioid analgesic, and has been used in clinics since 1917 [4]. Like other opioids, including morphine, the analgesic effect of oxycodone is mainly mediated through the activation of the μ-opioid receptor. Interestingly, the analgesic effect of oxycodone seems to be as potent as morphine on different types of pain, including visceral pain [5], post-operative pain [6] and cancer pain [7], [8]. Moreover, the oxycodone-paracetamol combination was shown to be effective against chronic lower back pain [9], whereas morphine treatment did not show any beneficial effect [10]. In patients with neuropathic pain, oxycodone treatment (mostly in combination with anticonvulsants) improves health-related quality of life and diminishes the impact of pain on physical activity and sleep [11]. However, the neurobiological mechanisms underlying the therapeutic action of these two opioids are currently only partially understood. The aim of this study was to identify the molecular changes that produce the different analgesic effects of morphine and oxycodone.

Vincristine is an anti-neoplasic drug used to treat a wide variety of cancers. Nonetheless, it induces a peripheral neurotoxicity and subsequent neuropathic pain in both patients [12], [13] and animal models [14]–[17]. Here, we observed in an animal model of vincristine-induced neuropathic pain that oxycodone has a longer lasting analgesic effect than morphine. Based on DNA microarray technology in dorsal root ganglia, we report an important list of genes that are differentially expressed in oxycodone-treated rats, as compared to morphine-treated subjects. This provides clues on the mechanisms underlying their different long-term actions. Furthermore, we provide direct evidence for an involvement of GABA_B_ receptors in the long-lasting effect of oxycodone on neuropathic pain.

## Materials and Methods

All experiments were performed in agreement with the European Community Council Directive of 24 November 1986 (86/609/EEC) and with guidelines on ethical standards for the investigation of experimental pain in animals, published in a Guest Editorial in the journal ‘Pain’ (Committee for Research and Ethical issues of the I.A.S.P., 1980). This study was approved by the ethics committee C2EA-59: ‘Comité d’éthique en matière d'expérimentation animale Paris Centre et Sud'. Accordingly to the rule of the ‘3R’ of the European regulation for the use of laboratory animals, the number of animals was kept to the minimum possible. Experiments were performed on 123 male Sprague Dawley rats (Janvier, Le Genest St Isle, France) weighing 150–175 g at the beginning of the experiments. Great care was taken, particularly with regard to housing conditions, to avoid or minimize discomfort of the animals: rats were housed four in a cage to minimize the possibility of painful interactions. The animals were kept on solid floor cages with a deep layer of sawdust to accommodate the excess of urination and cages were changed three times a week. They were kept at a constant temperature of 22°C, with a 12 h alternative light/dark cycle. Food and water were available *ad libitum*.

### Induction of peripheral neuropathy by vincristine and analgesic treatment

Animals were randomly chosen and tested by an experimenter blinded to the animal's treatment. Fifty-seven rats received intraperitoneal (i.p.) injections of vincristine (0.1 mg/kg/day; Oncovin-1 mg, EG LABO, Boulogne-Billancourt, France) for two 5-day cycles with a 2-day pause between cycles, as previously described [17]. Thirty control rats received i.p. injections of the vehicle (saline: 9‰ NaCl) according to the same protocol. At the end of vincristine treatment (D15), each group was divided into 3 sub-groups that received chronic injections of morphine (3.33 mg/kg diluted in saline, i.p. injection; laboratoire Cooper, France), oxycodone (3.33 mg/kg diluted in saline, i.p. injection; OxyNorm, Napp Pharmaceuticals Ltd, Cambridge, UK) or saline during 5 days (D19). These doses were chosen according to a previous report [18], as well as unpublished preliminary data showing an equipotent analgesic effect of morphine and oxycodone on mechanical hyperalgesia (pinch test) in naive animals, when used at 3.33 mg/kg.

### Influence of the GABA_B_ receptor on the long-lasting analgesic effect of repeated oxycodone injections: intrathecal administration of saclofen

This experiment was performed to evaluate the involvement of GABA_B_ receptors in the analgesic effect of oxycodone. Intrathecal injections were performed by lumbar puncture, as previously described [19], [20]. Briefly, a rat was slightly anesthetized with isoflurane gas (Baxter, Maurepas, France) and held in one hand by the pelvic girdle; a 25-gauge x1-inch needle connected to a 10-μl Hamilton syringe was then inserted into the subarachnoidal space between the spinous processes of L5 and L6, until a tail-flick was elicited. The syringe was held in position for several seconds after the injection of saclofen (10 μl; Ref S166, Sigma-Aldrich, St. Louis, MO) or saline. Saclofen was diluted to a concentration of 1 μg/μl in saline. This dose was chosen according to [21], [22]. Twenty-four rats received i.p. injections of vincristine. At D15, rats were subdivided into two groups: 12 rats received chronic injections of oxycodone, and the other 12 received chronic injections of saline (over a 5 day-treatment). At D19, each group was further divided into 2 sub-groups that received an i.t. injection of saclofen or saline (n = 6 vincristine-oxycodone-saclofen treated rats; n = 6 vincristine-oxycodone-saline treated rats; n = 6 vincristine-saline-saclofen treated rats; n = 6 vincristine-saline-saline treated rats).

### Influence of the GABA_B_ receptor on the analgesic effect of acute injection of morphine and oxycodone and repeated injections of morphine: intrathecal administration of saclofen

Thirty-six rats received i.p. vincristine injections. The role of the GABA_B_ receptor on the analgesic effect of acute injection of opioids was tested at D15 (n = 6 vincristine-oxycodone-saclofen treated rats; n = 6 vincristine-oxycodone-saline treated rats; n = 6 vincristine-morphine-saclofen treated rats; n = 6 vincristine-morphine-saline treated rats; n = 6 vincristine-saline-saclofen treated rats; n = 6 vincristine-saline-saline treated rats). The role of the GABA_B_ receptor on the analgesic effect of chronic injections of morphine was tested at D19, as with oxycodone (see above).

### Behavioural tests

The animals received a two-week habituation period prior to the start of the peripheral neuropathy-inducing vincristine injections, as previously described [23].

#### Chronic effects of analgesic treatment

Behavioural tests were performed on D1 and D15 of vincristine treatment in 21 vincristine-treated rats and 18 control rats. The time-course study on the effects of oxycodone and morphine was performed at D15 and D19, in which behavioural tests were repeated 30, 60 and 90 minutes following morphine (n = 7 vincristine-morphine treated rats; n = 6 saline-morphine treated rats), oxycodone (n = 7 vincristine-oxycodone treated rats; n = 6 saline-oxycodone treated rats), or saline injections (n = 7 vincristine-saline treated rats; n = 6 saline-saline treated rats).

#### GABA_B_ receptor antagonist study on the long-lasting analgesic effect of repeated injections of oxycodone

Behavioural tests were performed on D1 and D15 of vincristine treatment in 24 vincristine-treated rats. Behavioural tests were performed on D15 as a control for the development of neuropathic pain state; the animals were then repeatedly treated with oxycodone. The rats received one injection of oxycodone or saline at D19, and were then tested 30 minutes later as a control for the long-lasting analgesic effect of oxycodone (D19). Four hours after this first injection, rats were injected again with oxycodone or saline (D19′). Fifteen minutes after this second injection, rats were anesthetized with isoflurane (Baxter, Maurepas, France) and injected intrathecally with saclofen (10 μg; Ref S166, Sigma-Aldrich, St. Louis, MO) diluted in saline. Rats were behaviourally tested once again, 15 minutes after the saclofen injection (n = 6 vincristine-oxycodone-saclofen treated rats; n = 6 vincristine-oxycodone-saline treated rats; n = 6 vincristine-saline-saclofen treated rats; n = 6 vincristine-saline-saline treated rats).

#### GABA_B_ receptor antagonist study on the analgesic effect of acute injection of oxycodone or morphine and chronic injections of morphine

Behavioural tests were performed on D1 of vincristine treatment in 36 vincristine-treated rats. The rats received one injection of oxycodone, morphine or saline at D15. Fifteen minutes later, rats were anesthetized with isoflurane and injected intrathecally with saclofen (10 μg; Ref S166, Sigma-Aldrich, St. Louis, MO) diluted in saline. Rats were tested again 15 minutes after the saclofen injection (n = 6 vincristine-oxycodone-saclofen treated rats; n = 6 vincristine-oxycodone-saline treated rats; n = 6 vincristine-morphine-saclofen treated rats; n = 6 vincristine-morphine-saline treated rats; n = 6 vincristine-saline-saclofen treated rats; n = 6 vincristine-saline-saline treated rats). At D19 (*i.e*. 5 days after the start of opioids treatment), the same experiment was performed following chronic injections of morphine (n = 6 vincristine-morphine-saclofen treated rats; n = 6 vincristine-morphine-saline treated rats; n = 6 vincristine-saline-saclofen treated rats; n = 6 vincristine-saline-saline treated rats). For ease of reading, we have presented the results in two separate figures: the effect of saclofen after acute injection of oxycodone, and the effect of saclofen after morphine treatment.

#### Assessment of static mechanical sensitivity

The static mechanical allodynia was measured using the electronic Von Frey test, as previously described [23]. Briefly, an Electronic Von Frey hair unit (EVF-3, Bioseb, Chaville, France) was used to measure the sensitivity threshold in one test; measurements ranged from 0 to 500 grams with a 0.2 gram accuracy. Paw sensitivity threshold was defined as the minimal pressure required to elicit a robust and immediate withdrawal reflex of the paw. The stimulus was applied to each hind paw five times within a five-second interval; by convention, the threshold for each rat was the average of ten measured values. Static mechanical allodynia was defined as a significant decrease in withdrawal thresholds to Von Frey application.

The results for each group in the GABA_B_ receptor antagonist study were expressed as follows: δ-paw withdrawal threshold (%± S.E.M.), calculated from individual paw withdrawal thresholds: 
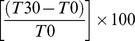
 at D19 and 
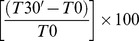
 at D19′. The effect of saclofen on mechanical allodynia was defined as a significant decrease in δ-paw withdrawal threshold to Von Frey application.

#### Assessment of static mechanical hyperalgesia

Static mechanical hyperalgesia was measured using the ‘Pinch’ test, as previously described [23]. Rats were individually taken by the experimenter and held without restraint on the experimenter's lap for 5 min. The threshold of static mechanical sensitivity was determined using a ‘Pincher’ unit (Bioseb, Chaville, France). Briefly, pressure was applied with a calibrated forceps (ranging from 0–800 g) to the mid-plantar area of each hind paw, to elicit a robust and immediate withdrawal reflex of the stimulated paw. The stimulus was applied to each hind paw three times within a 2 minute-interval; by convention, the threshold for a rat was the average of the six measured values. Static mechanical hyperalgesia was defined as a significant decrease in withdrawal thresholds to pincher application.

The results for each group in the GABA_B_ receptor antagonist study were expressed as follows: δ-paw withdrawal threshold (%± S.E.M.), calculated from individual paw withdrawal thresholds: 
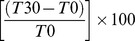
 at D19 and 
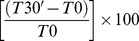
 at D19′. The effect of saclofen on mechanical allodynia was defined as a significant decrease in the δ-paw withdrawal threshold to pincher application.

#### Assessment of dynamic mechanical allodynia and hyperaesthesia: the “Paint-brush test”

The dynamic mechanical allodynia observed in neuropathic patients was measured as previously developed in our laboratory and described in Thibault *et al*. [24]. First, a stimulus was applied with a smooth (marten hair) paint-brush to each hind paw, five times within a five-second interval. Each stimulus that resulted in a response was scored as ‘1’, and the absence of a response was scored as ‘0’. The 10 stimulation scores were added, giving a total score between 0 and 10 for each rat. Next, the stimulation was applied with a rough (pig bristle) paint-brush, using the same protocol. Dynamic mechanical allodynia or hyperesthesia were defined as a significant increase in the total score using the smooth or the rough brush, respectively.

#### Statistical analysis of the behavioural data

Behavioural data from electronic Von Frey and Pinch tests were examined using two-way analysis of variance (ANOVA) followed by a Student Newman–Keuls test for two-by-two comparisons. Behavioural data from paint-brush tests were examined with a Kruskal–Wallis test followed by a Wilcoxon matches pairs test and a Mann–Whitney U test for two-by-two comparisons. Data were expressed as means ± SEM, and the levels of significance were set at: _*_p<0.05; _**_p<0.01; _***_p<0.001.

### DNA microarray analysis

#### RNA preparation for microarray analysis and microarray hybridisation

Two hours after the end of the behavioural study (D19), rats were deeply anaesthetized with pentobarbital (60 mg/kg i.p; Ceva Santé Animale, Libourne, France) and decapitated (n = 4 vincristine-morphine, n = 4 vincristine-oxycodone, n = 4 vincristine-saline treated rats; and n = 4 saline-morphine, n = 4 saline-oxycodone, n = 4 saline-saline rats). Lumbar dorsal root ganglia (DRGs) L2 through L5 were dissected and immediately frozen on dry ice. Tissues were kept at −80°C until homogenisation. Total RNA from individually dissected frozen DRGs was extracted and treated with DNAse using the RNeasy® Lipid Tissue Mini kit (Qiagen, Courtaboeuf, France), according to the manufacturer's protocol. The quality and quantity of each RNA sample was checked using an Agilent 2100 Bioanalyzer with RNA 6000 NanoChips (Agilent Technologies, Massy, France). Five hundred ng of RNA were sent to the Integragen Illumina microarray facility (Integragen, Evry, France, www.integragen.com) for sample processing. Illumina pangenomic rat microarrays (RatRef-12 Expression BeadChips), including 22,517 probes, were also performed at the Integragen Illumina microarray facility (Evry, France), according to the Illumina procedures.

#### DNA microarray statistical analysis

All manipulations and statistical analyses were performed with the R freeware. For Illumina beadchip data, all spots with a detection p-value greater than 0.05% were excluded. A two-way ANOVA was performed, using vincristine treatment as the first factor and analgesic treatment as the second factor. Student t-tests were performed to find differentially expressed genes between morphine and oxycodone treatment, in vincristine-treated rats or controls. The reported p-values correspond to the control of the type I error risk of each individual gene (no adjustment for multiple testing). The data have been deposited in NCBIs Gene Expression Omnibus (http://www.ncbi.nlm.nih.gov/geo/) and assigned Series accession number GSE53897.

#### Real-time quantitative PCR

Quantitative real-time PCR was performed using the fluorescent binding-dye SYBR Green (Absolute Blue SYBR Green Rox mix, ABgene, Thermo Scientific) on a real-time PCR machine (LightCycler Technology, Roche) with primer sets designed at the Roche website (www.roche-applied-science.com). Each qPCR assay was conducted in triplicate, using cDNA derived from 50 ng total RNA from each animal. Each sample well contained 4 μl of cDNA (5 ng/μl), with a primer concentration of 0.5 μM plus 5 μl of SYBR Green. Each qPCR assay was conducted with one candidate housekeeping gene for normalisation (actin, beta: Actb). The following primers were used: Gabbr2, 5′-AAGACCTTTGAAACACTCTGCAC-3′ (forward) and 5′-CCAAAGGCAGTTGTGTAGCC-3′ (reverse); Actb, 5′-CCCGCGAGTACAACCTTCT-3′ (forward) and 5′-CGTCATCCATGGCGAACT-3′ (reverse). qPCR was performed on all samples (saline-saline; saline-oxycodone; saline-morphine; vincristine-saline; vincristine-oxycodone; vincristine-morphine). All fluorescence data were processed using a post-PCR data analysis software program (LightCycler relative Quantification Software version 1.0; Roche), providing an automatic calculation of a normalized ratio. Saline samples were used for normalisation. The ratios of each gene of interest were compared among samples, using an unpaired t-test.

### Immunohistochemistry

#### Tissue preparation

Twelve rats receiving vincristine treatment and 12 others receiving saline treatment were used for the immunohistochemistry experiment. At D15, each group was divided into 3 sub-groups that received chronic injections of morphine (n = 4 vincristine-morphine, n = 4 saline-morphine treated rats), oxycodone (n = 4 vincristine-oxycodone, n = 4 saline-oxycodone treated rats) or saline (n = 4 vincristine-saline, n = 4, saline-saline treated rats). Rats were deeply anaesthetized at the end of analgesic treatment (D19) with a 25 mg/ml urethane injection (1.5 g/kg i.p), and perfused transcardially with 200 ml of 0.9% NaCl, followed by 500 ml of 4% paraformaldehyde (PFA) in 0.1 M phosphate buffer (PB), pH 7.4. Tissues were dissected out and post-fixed at 4°C in the same fixative, either overnight (spinal cord) or for 2 hours (DRGs). All tissues were further cryoprotected in 30% sucrose (in 0.1 M PB, pH 7.4) overnight at 4°C. DRG sections (14 μm thick) were serially cut using a cryostat (HM550, Microm Microtech). Sections were mounted directly onto a Superfrost slide such that two series of six slides corresponding to the whole ganglion could be obtained. Two slides per animal were used for each labelling. Spinal cord sections (30 μm thick) were cut using a cryostat and collected in phosphate buffer saline (PBS; 0.02 M) containing 0.02% sodium azide.

### Characterisation of the rabbit anti-GABAB2 antibody

#### Western Blot

The anti-GABA_B2_ antibody was tested using the western blot method. Briefly, a rat was deeply anaesthetized with pentobarbital (60 mg/kg i.p.; Ceva Santé Animale, Libourne, France) and then euthanised by decapitation. The cortex of a control animal was homogenised in RIPA (radioimmunoprecipitation assay buffer; 50 mM Tris, pH 8, 150 mM NaCl, 1% NP-40, 0.5% deoxycholate, 0.1% SDS, 1 mM sodium orthovanadate) containing Complete protease inhibitor mixture (Complete, Roche, Neuilly-sur-seine, France). The protein concentrations were determined using a Biorad DC Protein Assay kit (Bio-Rad Laboratories, California, USA). Spinal cord samples (30 μg of proteins) were separated by 10% SDS-PAGE, and transferred to nitrocellulose membranes. The membrane was then incubated with the primary antibody rabbit anti-GABA_B2_ (ref. 52248, Abcam, Cambridge, UK), diluted 1∶1000 in 20 mM Tris, pH 7.5, 500 mM NaCl, 0.1% Tween 20 (TBS-T). After several washes in TBS-T, the membrane was incubated in an anti-rabbit IgG HRP secondary antibody (1∶10000) for 1 h at room temperature. Following several washes in TBS-T, the membrane was treated with the ECL-plus reagent (10 min) for detection by autoradiography. A specific band was observed at 106-110 kDa, corresponding to the GABAB2 receptor (Figure S1A).

#### Immunohistochemistry

Immunostainings performed with this antibody on spinal cord revealed characteristic GABA_B2_ receptor staining. As previously reported [25], [26], we observed staining in the superficial laminae (Figure S1B) and in deeper laminae (Figure S1B). Spinal cord sections incubated without primary antibody did not display any staining (data not shown).

### GABAB2 receptor labelling in dorsal root ganglia

Immunostainings were performed on tissues from 4 animals per group. Double immunofluorescent stainings for GABA_B2_ receptor and βIII tubulin (a marker of all neuronal cells) were performed to determine the percentage of neurons expressing GABA_B2_ receptors. Sections were incubated overnight at room temperature with a rabbit anti-GABA_B2_ receptor antibody (1∶300, ref. 52248, Abcam, Cambridge, UK) and mouse anti-βIII Tubulin antibody (1∶2000, ref. G7121, Promega, San Luis Obispo, CA USA); antibodies were diluted in PBS-T-azide (PBS 0.02 M containing 0.3% Triton X-100 and 0.02% sodium azide). Following several washes in PBS, sections were incubated for 2 h at room temperature in Alexa Fluor® 488 anti-rabbit IgG and Alexa Fluor® 350 anti-mouse IgG (Invitrogen, California, USA), diluted 1∶1000 in PBS-T. Following several washes in PBS, sections were mounted in Vectashield medium (Vector Laboratories, Burlingame, USA). A similar procedure was performed for GABA_B2_ receptor-GABA_B1_ receptor double immunostaining, using the anti-GABA_B1_ receptor antibody (1∶500; mouse anti-GABA_B1_ receptor, ref. 55051, Abcam, Cambridge, UK) and Alexa Fluor® 546 anti-mouse IgG.

#### Quantification of βIII tubulin- and GABAB2 receptor-positive neurons

Black and white 256 grey level images were acquired using a 40X objective on a Zeiss microscope equipped with a Zeiss Axiocam MRm camera. All acquisitions were performed using the same acquisition setup. Quantification of the percentage of GABA_B2_ receptor-positive cells was performed by counting both the number of GABA_B2_ and βIII tubulin immunopositive cells in DRG. At least 200 cells were counted per DRG (more than four or five sections per rat). An analysis of staining intensity and size diameter distribution was performed using Image J software (http://rsb.info.nih.gov/ij/). The background staining intensity was subtracted from each image, and results were expressed as the mean intensity of immunoreactivity ± SEM. The mean diameter, percentage of positively stained cells, and mean intensity of GABA_B2_ staining in DRG neurons were compared between all groups by one-way ANOVA using SigmaStat software, followed by the Student Newman–Keuls test to detect differences between different groups.

#### GABAB2 receptor labelling in the spinal dorsal horn

Free floating serial sections from the lumbar segment L4-L5 were incubated in rabbit anti-GABA_B2_ receptor antibody (1∶300; ref. 52248, Abcam, Cambridge, UK). This was either performed alone for quantification, or in combination with the following antibodies for double or triple staining: 1) IB4, a FITC-conjugated isolectine (1∶700; Isolectin B4 FITC conjugate, ref. L 2895, Sigma-Aldrich, St. Louis, MO); 2) mouse anti-CGRP (1∶500; ref. C 9487, Sigma-Aldrich, St. Louis, MO); 3) guinea pig anti-vGlut2 (1∶5000; ref. AB2251, Chemicon, Temecula, CA USA); or 4) mouse anti-GABA_B1_ receptor (1∶500; ref. 55051, Abcam, Cambridge, UK, [27]). Tissues were finally washed in PBS, and mounted in Vectashield medium (Vector, Burlingame, USA).

#### Quantification of GABAB2 receptor labelling in the spinal dorsal horn

To quantify GABA_B2_ receptor staining in the dorsal horn of all subject groups, black and white 256 grey level images were acquired using a 40X objective on a Zeiss microscope equipped with a Zeiss Axiocam MRm camera. All acquisitions were performed using the same acquisition setup. A 100 μm ×500 μm region of interest was placed on the dorsal horn to measure fluorescence intensity (FI) plot profiles (gray levels, in arbitrary units, with respect to the distance from the surface, in micrometers). Statistical significance was determined with a Student's unpaired t test for specific depth point, or one way ANOVA followed by a Student Newman–Keuls test for mean fluorescent intensity analysed per lamina (for all statistical tests: **p<0.01;*p<0.05).

As a final note, each type of immunostaining in the DRG or dorsal horn was simultaneously performed for all animals, in order to maintain consistency and allow a statistical analysis of all results. Omission of the primary antibody, or any other stage in the protocol, did not result in any labelling.

## Results

### Oxycodone maintained a strong analgesic effect after chronic treatment as compared to morphine in vincristine-treated rats

**Figure 1:**
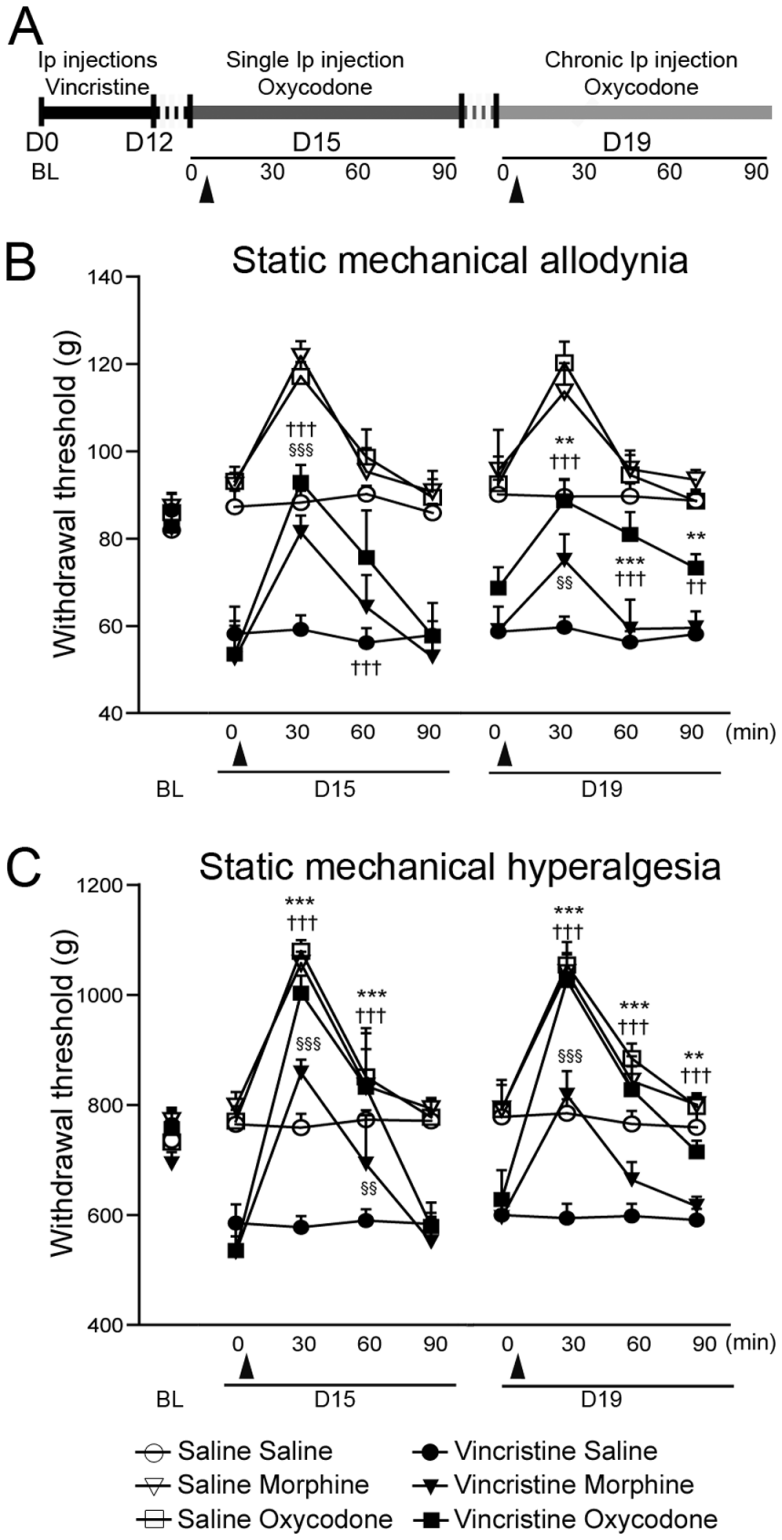
Time-course of static mechanical sensitivity. A: Schematic representation of the experimental paradigm. Rats received i.p. injections of vincristine (0.1 mg/kg/day) during 2 cycles of 5 days. Analgesic treatment started at D15, and was performed for 5 days until D19. The analgesic effect of oxycodone or morphine was tested on D15 and D19, with a time-course of 30, 60 and 90 minutes after injection. These injections are illustrated by black triangles. At D15, all groups of vincristine-treated rats presented static mechanical allodynia (B) and hyperalgesia (C), as compared to control rats and the baseline (BL). B: Static mechanical allodynia measured using the electronic Von Frey test. The measured static mechanical allodynia was totally reversed after a single injection of morphine or oxycodone, although only oxycodone maintained a strong analgesic effect at D19. C: Static mechanical hyperalgesia measured by the “Pinch Test”. Oxycodone induced an analgesic effect in vincristine-oxycodone treated rats after a single injection, and maintained its analgesic effect at D19. All data are expressed as mean ± SEM. (n = 7 vincristine-morphine treated rats; n = 6 saline-morphine treated rats), (n = 7 vincristine-oxycodone treated rats; n = 6 saline-oxycodone treated rats), and (n = 7 vincristine-saline treated rats; n = 6 saline-saline treated rats). *p<0.05, **p<0.01, ***p<0.001: vincristine-oxycodone treated rats vs. vincristine-morphine treated rats; † p<0.05, †† p<0.01, ††† p<0.001: vincristine-oxycodone treated rats vs. vincristine-saline treated rats; §§ p<0.01, §§§ p<0.001: vincristine-morphine treated rats vs. vincristine-saline treated rats. For figure clarity, the statistical significance symbols for the vincristine vs. saline groups are not shown.

As previously observed [28], at the end of vincristine treatment (D15), vincristine-treated rats displayed a significant increased static (Figure 1B-C) and dynamic (Figure S2A–B) mechanical sensitivity, as compared to the baseline (BL, *i.e*. beginning of the treatment) and saline-treated rats. A single injection of morphine or oxycodone was capable of totally reversing static mechanical allodynia, hyperalgesia (D15 T30, Figures 1B and C, respectively) and dynamic mechanical allodynia (D15 T30, Figure S2A). The analgesic effect of oxycodone (measured by the Pinch test) was more potent than the analgesic effect of morphine on static mechanical hyperalgesia (D15 T30, Figure 1C). Moreover, dynamic mechanical hyperesthesia (measured with the rough paintbrush test) was only attenuated by morphine, whereas it was totally reversed by oxycodone (D15 T30, Figure S2B). At the end of the analgesic chronic treatment on day 19 (D19), only oxycodone was able to maintain the analgesic effect observed at D15 on mechanical sensitivity (D19 T30, Figure 1B-C and Figure S2A–B). By this point, morphine had completely lost its analgesic effect on dynamic mechanical sensitivity (Figure S2A–B).

### Extensive analysis of gene expression modified by chronic analgesic treatment

We conducted a large-scale DNA microarray study based on pan-genomic chips, using Illumina technology on the dorsal root ganglion (DRG). This was aimed at systematically detecting genes whose expression was altered by chronic morphine or oxycodone treatment in either saline- or vincristine-treated rats. Among the 22,517 probes included in the microarray, 757 were differentially expressed by the end of analgesic treatment (D19), corresponding to 316 genes (Table S1). The complete list of dysregulated genes is catalogued in Table S1. Among these results, we report 145 genes that are differentially expressed after analgesic treatment in control rats (Table S1). This list could be applied to improve our understanding of the molecular mechanisms underlying chronic opioid treatment in steady state, and in particular the mechanisms of tolerance observed after chronic morphine treatment.

In this study, we chose to investigate the molecular mechanisms controlling the sustained analgesic effect of oxycodone in neuropathic pain, as compared to morphine. Our analysis was focused on genes differentially expressed after analgesic treatment in vincristine-treated rats; we thus identified 37 genes (Table S1). The difference in gene expression between vincristine-morphine and vincristine-oxycodone conditions was calculated (Table 1), revealing 15 genes whose expression was up-regulated after morphine treatment; this can also be expressed as (vincristine-morphine gene expression) - (vincristine-oxycodone gene expression) >0. Furthermore, we identified 22 genes whose expression was up-regulated after oxycodone treatment; this can also be expressed as (vincristine-morphine gene expression) - (vincristine-oxycodone gene expression) <0 (Table 1). Among the genes up-regulated after oxycodone treatment, we observed 3 genes that regulate receptor activity, classified in the ‘Receptor’ subcategory. Interestingly, these genes were: i) Gabbr2, coding for the GABA_B2_ receptor; and ii) Gabrb3 and Gabrg1, coding respectively for the GABA_A_ R subunit β3 and GABA_A_ R subunit γ1 (Table 2). The differential expression of these three genes was significant between morphine- and oxycodone-treated rats after vincristine treatment.

**Table 1:** List of genes differentially regulated between vincristine-morphine vs. vincristine-oxycodone treated animals.

								Variance analysis Vin vs Control	Mor vs Oxy Vincritsine animals		Mor vs Oxy Control animals	
ACCESSION	SYMBOL	V-Mor	V-Oxy	V-NaCl	S-Mor	S-Oxy	S-NaCl	p	p	diff	p	diff
NM_144737	Fmo2	7,19	7,67	7,27	7,01	7,03	7,01	0,01	0,04	−0,48	0,92	−0,02
NM_145670	Bcas1	8,88	9,22	8,95	9,16	8,81	9,15	0,77	0,01	−0,34	0,01	0,35
NM_031345	Dsipi	11,74	12,02	11,78	11,86	11,83	11,71	0,52	0,04	−0,28	0,82	0,03
NM_012829	Cck	6,26	6,53	6,29	6,34	6,31	6,31	0,48	0,01	−0,27	0,79	0,03
NM_134398	P34	6,06	6,28	6,22	6,24	6,16	6,24	0,57	0,01	−0,22	0,26	0,08
NM_053349	Sox11	6,02	6,24	6,09	6,12	6,12	6,05	0,8	0,02	−0,22	0,98	0
NM_013066	Mtap2	6,16	6,39	6,33	6,29	6,18	6,35	0,73	0,03	−0,22	0,27	0,11
NM_001004132	Pctk1	8,5	8,71	8,54	8,84	8,73	8,7	0,01	0,04	−0,21	0,28	0,1
NM_203367	Zmynd11	6,2	6,4	6,48	6,43	6,34	6,41	0,5	0,02	−0,2	0,28	0,09
NM_001006985	Mrpl13	10,81	11,01	10,97	10,97	10,92	10,92	0,95	0,02	−0,2	0,46	0,06
NM_053555	Vamp5	8,05	8,25	8,18	8,15	8,13	8,12	0,62	0,03	−0,2	0,85	0,02
NM_031613	Tmod2	7,74	7,94	7,82	7,81	7,82	7,93	0,68	0,04	−0,19	0,93	−0,01
**NM_031802**	**Gabbr2**	**11,15**	**11,34**	**11,17**	**11,19**	**11,19**	**11,22**	**0,64**	**0,04**	**−0,19**	**0,99**	**0**
NM_012839	Cycs	12,27	12,44	12,4	12,33	12,22	12,33	0,11	0,04	−0,18	0,2	0,11
NM_134351	Mat2a	6,27	6,45	6,4	6,36	6,19	6,39	0,11	0,01	−0,18	0,01	0,17
XM_343564	Col5a2	9,93	10,1	10,12	10,14	9,93	10,18	0,45	0,04	−0,17	0,02	0,21
NM_001005884	Letm1	8,99	9,14	9,12	9,05	9,15	9,04	1	0,04	−0,15	0,15	−0,1
NM_080577	Nploc4	6,09	6,24	6,18	6,18	6,12	6,11	0,35	0,03	−0,14	0,38	0,05
NM_053527	Cdc5l	9,2	9,34	9,34	9,38	9,18	9,32	0,98	0,03	−0,14	0	0,2
**NM_080586**	**Gabrg1**	**6,16**	**6,29**	**6,33**	**6,26**	**6,2**	**6,24**	**0,43**	**0,04**	**−0,13**	**0,31**	**0,06**
**NM_017065**	**Gabrb3**	**5,95**	**6,07**	**6,01**	**6,07**	**6,05**	**6,06**	**0,08**	**0,01**	**−0,12**	**0,59**	**0,02**
NM_053483	Kpna2	9,33	9,45	9,41	9,4	9,49	9,41	0,28	0,04	−0,12	0,1	−0,1
NM_001004206	Pa2g4	6,03	5,92	5,98	6	5,99	5,96	0,8	0,01	0,1	NaN	NaN
NM_171993	Cdc20	6,43	6,32	6,35	6,38	6,55	6,44	0,01	0,04	0,11	0,01	−0,17
NM_053854	Nat2	6,04	5,92	6,03	6,01	6,12	6,04	0,05	0,02	0,11	0,02	−0,11
NM_031129	Tceb2	11,07	10,95	11	11,02	11,02	10,93	0,54	0,04	0,12	0,99	0
NM_021657	Phlpp	8,19	8,07	8,09	8,21	8,21	8,2	0,01	0,03	0,12	0,96	0
NM_133553	B3galt4	6,42	6,26	6,42	6,41	6,39	6,41	0,3	0,02	0,16	0,67	0,03
NM_058208	Socs2	6,33	6,13	6,1	6,12	6,19	6,23	0,85	0	0,2	0,24	−0,07
NM_017030	Pccb	6,7	6,5	6,6	6,66	6,57	6,66	0,48	0,01	0,2	0,25	0,09
NM_001004259	Pnkp	6,6	6,39	6,51	6,52	6,55	6,46	0,78	0,01	0,21	0,63	−0,03
NM_053719	Emb	8,22	8	8,16	8,03	8,11	8	0,15	0,03	0,22	0,38	−0,09
NM_001007626	Ggps1	7,43	7,18	7,26	7,2	7,34	7,24	0,61	0,02	0,24	0,16	−0,14
NM_030834	Slc16a3	6,94	6,66	6,76	6,73	6,72	6,6	0,19	0,04	0,28	0,92	0,01
NM_001007612	Ccl7	6,51	6,21	6,57	6,5	6,51	6,45	0,44	0,03	0,3	0,91	−0,01
NM_012947	Eef2k	8,77	7,65	8,67	7,65	8,92	9,18	0,48	0,04	1,12	0,03	−1,27
NM_031010	Alox15	10,66	9,43	10,22	6,43	6,29	6,38	0	0,01	1,22	0,74	0,13

Data are illustrated as follows: 1) Gene accession number; 2) Gene symbol; 3) Mean expression of the genes for the six different groups; 4) results of variance analysis between vincristine and control: p-value; 5) results of Student's t-test between morphine and oxycodone in vincristine-treated rats: p-value and expression difference (morphine – oxycodone); 6) results of Student's t-test between morphine and oxycodone in control rats: p-value and expression difference (morphine – oxycodone).

**Table 2:** Modification of genes regulating GABA receptor activity expression after oxycodone treatment.

	GABA_A_ R subunit β3	GABA_A_ R subunit γ1	GABA_B2_ R
**Mean Expression**			
Vincristine-morphine	5.95±0.05	6.16±0.10	11.15±0.08
Vincristine-oxycodone	6.07±0.09	6.29±0.04	11.34±0.12
Vincristine-saline	6.01±0.02	6.33±0.10	11.17±0.13
Control-morphine	6.07±0.03	6.26±0.07	11.19±0.12
Control-oxycodone	6.05±0.02	6.20±0.11	11.19±0.14
Control-saline	6.06±0.09	6.24±0.03	11.22±0.16
**Statistics**			
p-value vincristine vs. control	0.081	0.429	0.644
Difference of expression	−0.048	0.027	0.024
**p-value morphine vs. oxycodone in vincristine animals**	**0.012**	**0.038**	**0.047**
**Difference of expression**	**−0.118**	**−0.129**	**−0.192**
p-value morphine vs. oxycodone in control animals	0.591	0.312	0.992
Difference of expression	0.023	0.060	−0.001

Data are illustrated as follows: 1) Mean expression value for all groups; 2) Statistical analysis: results of variance analysis between vincristine and control: p-value and expression difference (vincristine – control); results of Student's t test between morphine and oxycodone in vincristine-treated rats: p-value and expression difference (morphine – oxycodone); results of Student's t test between morphine and oxycodone in control rats: p-value and expression difference (morphine – oxycodone). The three genes regulating GABA receptor activity expression were exclusively modified after analgesic treatment in vincristine-treated animals.

Although we could not exclude a role of the GABA_A_ receptor in chronic opioid treatment, as reported in previous studies [29], [30], we chose to concentrate the second part of this study on GABA_B2_ receptor expression, and their potential role in the long-lasting analgesic effect of oxycodone on neuropathic pain.

### Chronic oxycodone treatment induced an increase in GABA_B2_ receptor expression in small diameter DRG neurons, as well as their terminals in superficial laminae of the dorsal horn

**Figure 2:**
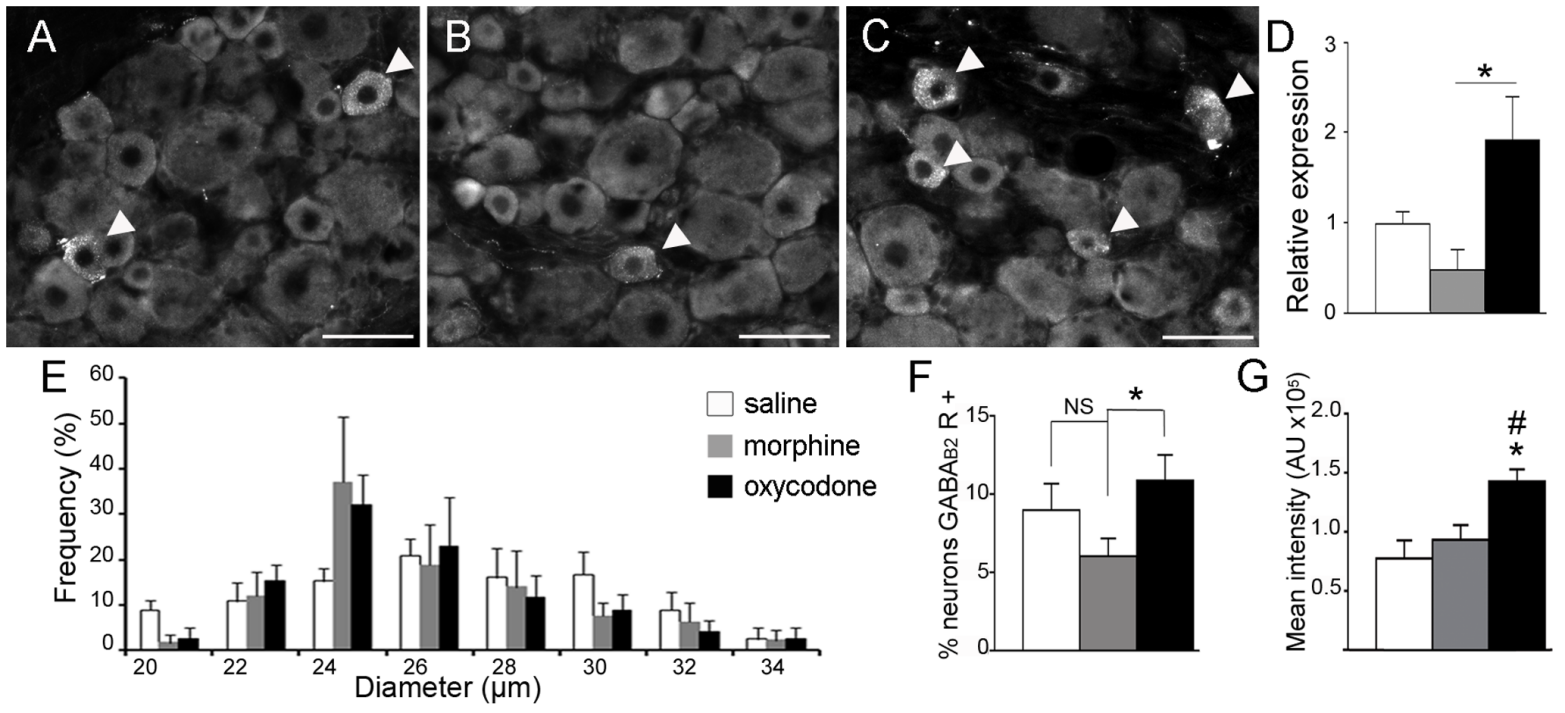
Up-regulation of GABA_B2_ receptor expression in small diameter DRG neurons after chronic oxycodone treatment. A–C: Immunofluorescent staining showing GABA_B2_ receptor immunoreactive neurons (arrowheads) in representative vincristine-saline (A), vincristine-morphine, (B) and vincristine-oxycodone (C) treated animals. Scale bars  = 50 μm. D: Relative expression of Gabbr2 in the DRG of vincristine-treated rats determined by qPCR. The results validate the up-regulation of the Gabbr2 gene observed by DNA microarray in the vincristine-oxycodone treated rat, as compared to a vincristine-morphine treated rat. Data obtained from vincristine-treated rats were normalized to control rat data (n = 4 per group; *p<0.05 between vincristine-oxycodone treated rats and vincristine-morphine treated rats). Data were expressed as mean ± SEM. E: Histogram showing the size distribution frequency of GABA_B2_ receptor expression in the DRG for all vincristine groups. Note that the size distribution frequency was identical in the three groups studied. F: Percentage of GABA_B2_ receptor-positive neurons in the three animal groups. Oxycodone induced an increased percentage of GABA_B2_ receptor-positive neurons as compared to morphine treatment. G: Analysis of the mean intensity of GABA_B2_ receptor staining in the three vincristine-treated animals. Oxycodone induced a 53% increase in GABA_B2_ receptor staining in vincristine-oxycodone treated rats as compared to vincristine-morphine treated rats, and an 84% increase as compared to vincristine-saline treated rats. (n = 4 vincristine-morphine treated rats, n = 4 vincristine-oxycodone treated rats, and n = 4 vincristine-saline treated rats) *p<0.05 between vincristine-oxycodone treated rats and vincristine-morphine treated rats; #p<0.05 between vincristine-oxycodone treated rats and vincristine-saline treated rats. Data are expressed as mean ± SEM.

In order to validate our results, we verified the differential expression of the GABA_B2_ receptor (Gabbr2) in the DRG of vincristine-treated and control animals at the RNA and protein levels. Using qPCR, we observed an up-regulation of Gabbr2 expression in vincristine-oxycodone treated animals as compared to vincristine-morphine treated animals (1.91±0.48 *vs*. 0.47±0.24; p<0.05) (Figure 2D). At the protein level, GABA_B2_ receptors were exclusively expressed in small and medium diameter neurons in DRGs (size ≤34 μm, Figures 2A-C and E). The size distribution histogram of GABA_B2_ receptor immunoreactivity in DRG cells was similar in all animal groups, with a peak at 24 μm (Figure 2E). However, we observed an increase in the percentage of neurons positive for GABA_B2_ staining in oxycodone-treated animals as compared to morphine-treated animals, following vincristine treatment (Figure 2F). Moreover, quantification of the mean intensity of GABA_B2_ receptor staining showed a 53% increase in vincristine-oxycodone treated rats as compared to vincristine-morphine treated rats, and an 84% increase compared to vincristine-saline treated rats (Figure 2G).

**Figure 3:**
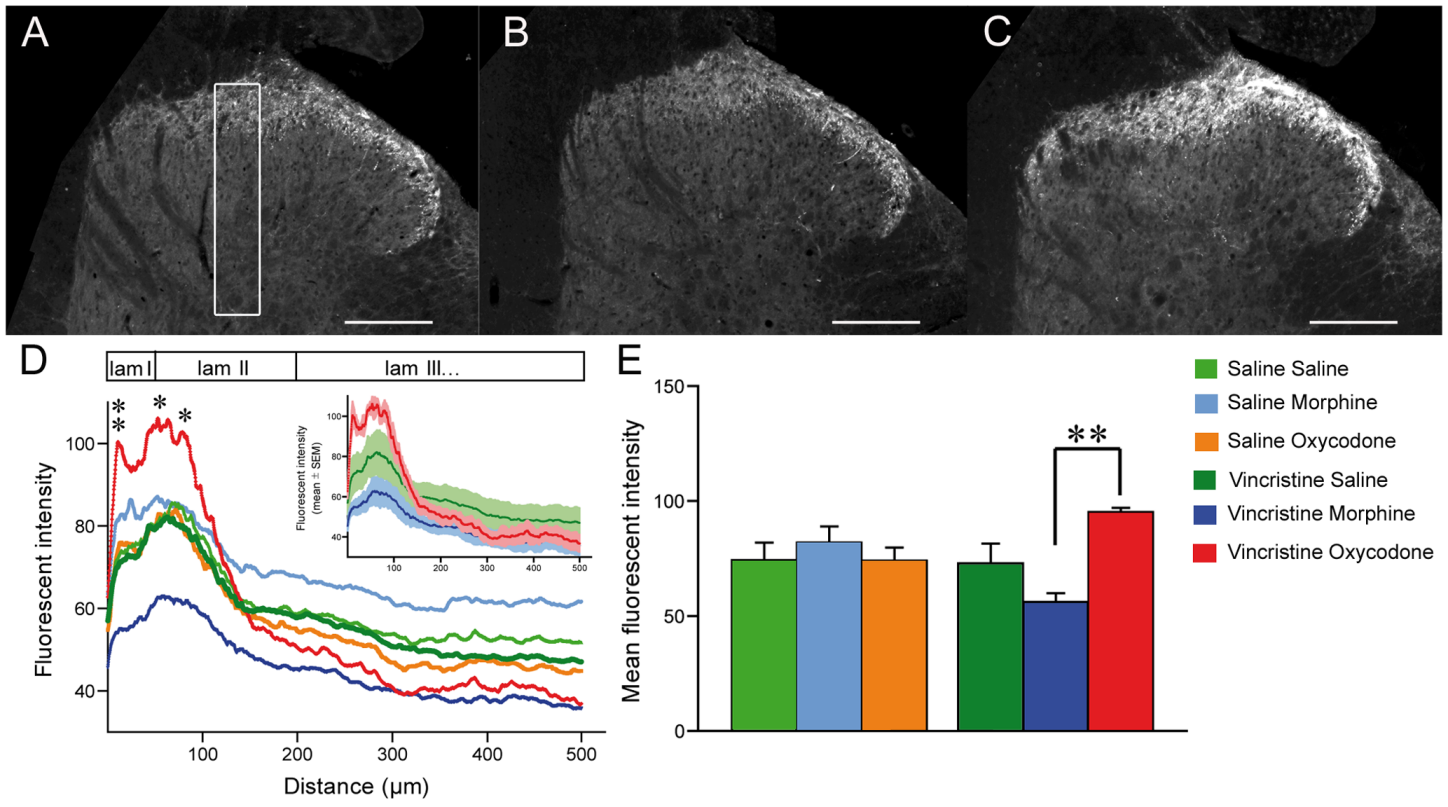
Up-regulation of GABA_B2_ receptor expression in superficial laminae of the spinal cord after chronic oxycodone treatment. A–C: Immunofluorescent staining showing GABA_B2_ receptor labelling in the dorsal horn in (A) vincristine-saline, (B) vincristine-morphine, and (C) vincristine-oxycodone treated animals. D: Analysis of the fluorescence intensity profiles of GABA_B2_ receptor immunoreactivity from superficial to deeper layers in control/vincristine treated animals chronically treated with saline, morphine or oxycodone. The selected zone was a vertical area in laminae I through IV (white selection in A). The insert shows the mean fluorescent intensity ± SEM in the three groups of vincristine-treated rats as mean ± SEM. For ease of reading, it was not possible to show the SEM of all groups. E: Mean fluorescent intensity of GABA_B2_ receptor immonoreactivity at the level of the first 50 μm of the spinal cord dorsal horn. Vincristine treatment did not alter the intensity of GABA_B2_ receptor staining as compared to saline-treated animals. However, oxycodone (but not morphine) significantly increased the intensity of GABA_B2_ receptor staining in the superficial lamina. (n = 4 vincristine-morphine treated rats; n = 4 saline-morphine treated rats), (n = 4 vincristine-oxycodone treated rats; n = 4 saline-oxycodone treated rats), and (n = 4 vincristine-saline treated rats; n = 4 saline-saline treated rats) *p<0.05 and **p<0.01 vincristine-oxycodone vs. vincristine-morphine. Data are expressed as mean ± SEM.

In the spinal dorsal horn, the intensity of GABA_B2_ staining exhibited a significant increase in both lamina I and the outer part of lamina II (Figure 3A–D), with a 70% increase in lamina I only in the vincristine-oxycodone treated group (Figure 3E). In contrast, there was no significant change in the other groups, or in deep laminae (Figure 3D).

### Functionality and localisation of GABA_B2_ receptors

**Figure 4:**
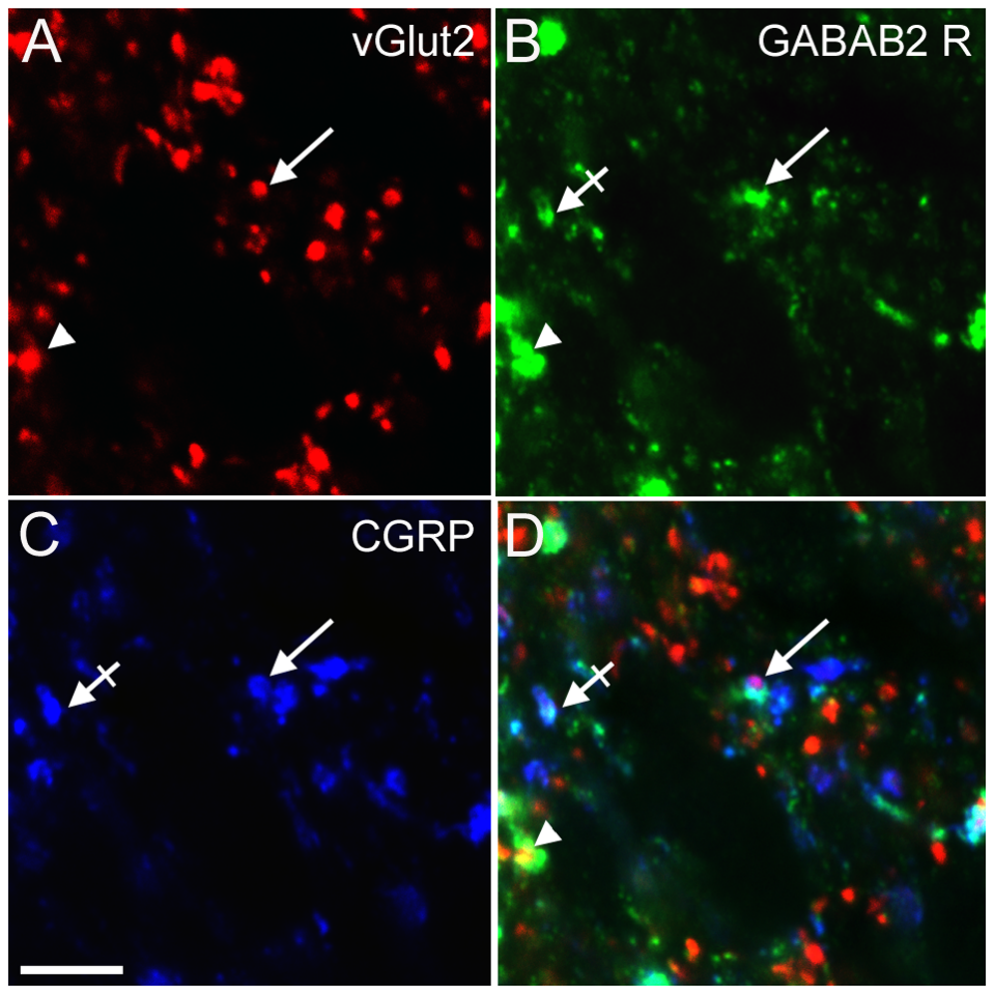
The GABA_B2_ receptor is located in CGRP-positive glutamatergic presynaptic terminals. Confocal images of triple immunostainings, comprised of vGlut2 (A, red), GABA_B2_ receptor (B, green) and CGRP (C, blue), in a vincristine-oxycodone treated animal. D is a merge of images A–C. We observed GABA_B2_ receptor expression in CGRP-immunoreactive glutamatergic presynaptic terminals (arrow). Glutamatergic presynaptic terminals immunoreactive for GABA_B2_ receptor were also observed (arrowhead), as well as CGRP-positive GABA_B2_ terminals (crossed arrow). Scale bar A-D = 5 μm.

To be functional, GABA_B_ receptors must be organised as heterodimers comprising one GABA_B1_ and one GABA_B2_ subunit (for review see [31]). Using double immunostaining, we effectively observed their colocalisation in small and medium sized DRG neurons (Figure S3A–C) and in superficial layers of the dorsal horn (Figure S3D–F), suggesting that functional GABA_B_ receptors are expressed at these levels. In addition, we observed that GABA_B2_ receptors mainly colocalised within peptidergic CGRP-positive fibres (Figure S4A–C; Figure 4B–C–D, crossed arrows), and not in non-peptidergic IB4-positive terminals (Figure S4D–F). Notably, we observed that GABA_B2_ receptors were present in glutamatergic presynaptic terminals (vGlut2 positive) containing CGRP (Figure 4A–D, arrows).

**Figure 5:**
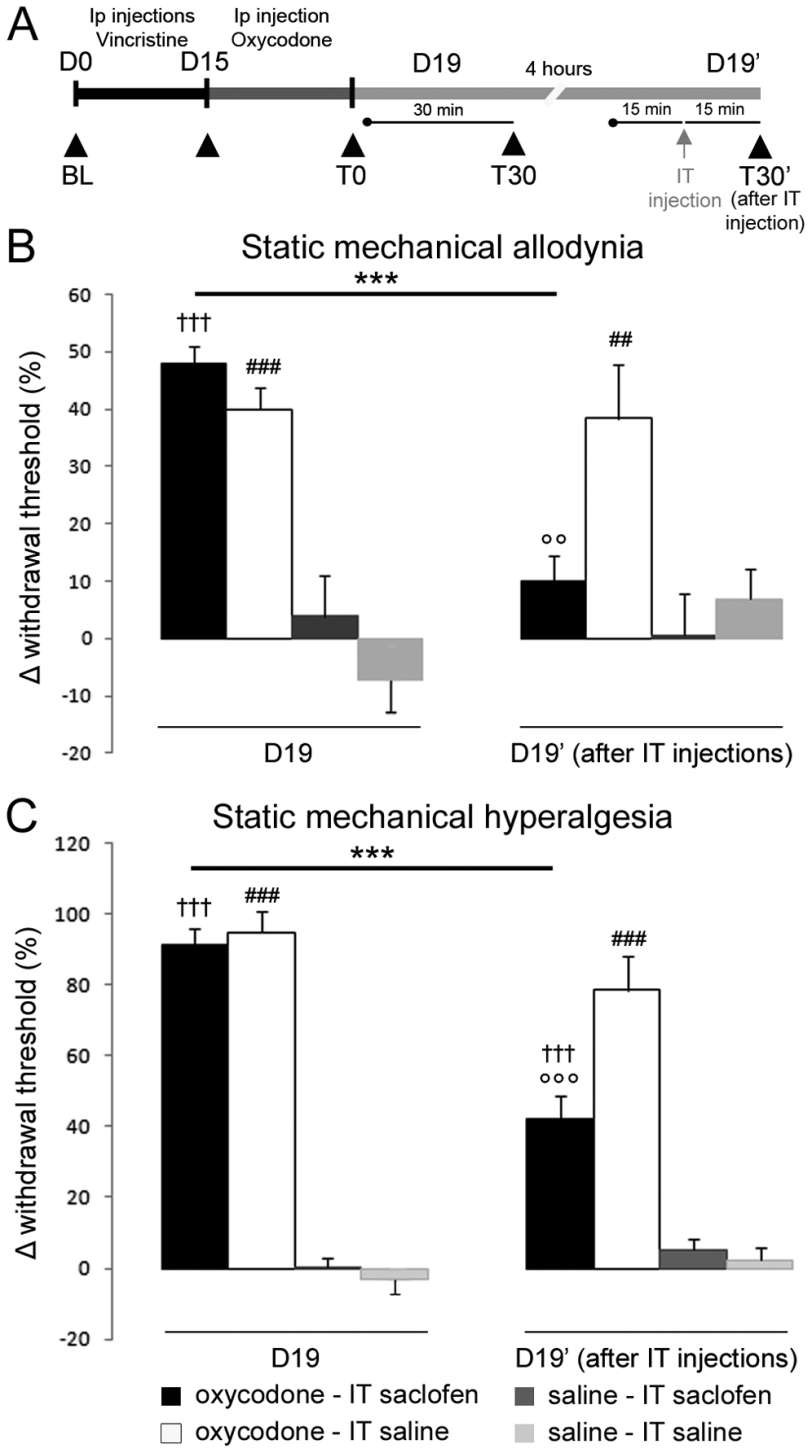
The long-lasting analgesic effect of oxycodone is reversed by i.t. injection of a GABA_B_ antagonist. A: Schematic representation of the experimental paradigm. Rats received i.p. injection of vincristine (black horizontal bar). The oxycodone treatment started at D15, while animals presented mechanical hyperalgesia (dark grey horizontal bar). Behavioural tests, illustrated by black triangles, were performed at D0 (BL), D15 and D19, before (T0) and 30 min after (T30) oxycodone injection, to control for the analgesic effect of oxycodone. The functional involvement of GABA_B_ receptors in this long-asting analgesic effect of oxycodone was tested by intrathecal (i.t.) injection of saclofen (10 μg) versus saline injection (grey arrow), 4 hours after the previous test (D19). Rats were injected again with oxycodone or saline (D19′). Fifteen minutes after this second injection, rats were injected intrathecally with saclofen or saline and tested 15 minutes after the i.t. injection (T30′). B, C: Effect of saclofen intrathecal injection on oxycodone's long-lasting effect on static mechanical allodynia (B) and static mechanical hyperalgesia (C). Results are expressed as mean δ withdrawal threshold ± SEM. The δ-withdrawal threshold was calculated using individual values as follows: 
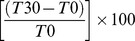
 at D19 and 
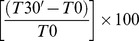
 at D19′. The results confirm the analgesic action of oxycodone (left panel, D19), and show that saclofen completely reversed the analgesic action on mechanical allodynia (B), while only partially reversing the effect on static mechanical hyperalgesia (C). (n = 6, vincristine-oxycodone-saclofen treated rats; n = 6, vincristine-oxycodone-saline treated rats; n = 6 vincristine-saline-saclofen treated rats; n = 6, vincristine-saline-saline treated rats). ***p<0.001: vincristine-oxycodone treated rats compared at different time points; ## P<0.01, ### p<0.001: vincristine-oxycodone treated rats vs. vincristine-saline treated rats; °°p<0.01, °°°p<0.001: vincristine-oxycodone-saclofen treated rats vs. vincristine-oxycodone-saline treated rats; ††† p<0.001: vincristine-oxycodone-saclofen treated rats vs. vincristine-saline-saclofen treated rats.

We directly examined the role of GABA_B_ receptors in the long-lasting analgesic effect of oxycodone by assessing the effect of intrathecal administration of saclofen (10 μg), a selective GABA_B_ receptor antagonist [21], [22] (Figure 5A). The relieving effect of oxycodone on static mechanical allodynia was totally blocked by the antagonist (Figure 5B), whereas its analgesic effect on mechanical hyperalgesia was only partially blocked (Figure 5C). Interestingly, intrathecal administration of saclofen had no effect on the analgesic action of oxycodone after a single injection at D15 (Figure S5A–B). Moreover, in order to demonstrate the specific involvement of up-regulated GABA_B_ receptor function in the long-lasting analgesic effect of chronic oxycodone injection, we assessed the effect of an intrathecal administration of saclofen after a single injection (D15) and after chronic administration of morphine (D19). These results indicate that saclofen does not inhibit the effect of acute or chronic treatment of morphine on mechanical sensitivity in vincristine-treated rats (Figure S6A–B).

## Discussion

The aim of this study was two-fold: to better understand the mechanisms underlying the analgesic effect of both oxycodone and morphine; and to compare these mechanisms of action in a model of neuropathic pain induced by vincristine. Our results demonstrate that a large number of genes are dysregulated after opioid analgesic treatment in vincristine-treated animals. These findings also suggest an increased GABAergic tone in the superficial dorsal horn induced by oxycodone treatment that is mediated by GABA_B2_ receptor; this would explain the enhanced alleviation of chronic neuropathic pain that we observed.

### Attenuation of the analgesic effect of morphine

Vincristine-treated rats displayed all of the behavioural characteristics of peripheral sensory neuropathy observed in this model, *i.e*. static and dynamic mechanical allodynia and hyperalgesia [15], [17], [24], [32]. As previously described in both patients and animal models [2], [3], [5], [8], , we observed that administration of oxycodone or morphine alleviated symptoms of chronic neuropathic pain. However, repeated morphine injections lead to an attenuation of its analgesic effect, while oxycodone maintained a strong analgesic effect even after multiple injections. These observations are consistent with previous reports demonstrating morphine's inefficacy in the symptomatic relief of neuropathic pain in STZ-induced diabetes models [36], [37], as well as an observed slower rate of tolerance to oxycodone-6-oxyme (an oxycodone derivative compound) as compared to morphine in naive animals [18]. While morphine and oxycodone are both μ-opioid receptor agonists, previous studies have shown that these two opioids exhibit different efficiencies and distinct analgesic profiles in various pain models, such as the bone cancer pain model and the SNL model [33]. A more recent study from that same group has shown that modification of the μ-opioid receptor is responsible for the distinct analgesic effect of oxycodone and morphine, and that μ-opioid receptor activation is less attenuated by oxycodone than by morphine [38]. Although the authors of that study dismiss the hypothesis that, in the bone cancer pain model, a change in the number of μ-opioid receptors or a change in the μ-opioid receptor binding affinity might be the underlying mechanisms, these hypotheses remain valid in our model. Finally, as proposed by Nakamura *et al*., the differential activation of the μ-opioid receptor by oxycodone or morphine might be due to the mechanism that regulates GTP binding onto G proteins in the μ-receptor [38]. These changes in μ-opioid receptor activation might produce subtle but functionally important variations in intracellular cascade signalling, possibly leading to a modified gene expression that is observed after oxycodone treatment, but not after morphine treatment.

Previous studies have reported that repeated opiate administration alters gene expression in different regions of the nervous system in rodents [29], [30], [39], which may contribute to plastic changes associated with tolerance. It has also been shown that morphine treatment alters the expression of several receptors in the amygdala, including the GABA_A_ receptor [29]. Moreover, chronic treatment of morphine leads to a decrease in GABAergic tonus [40], [41]. Our DNA microarray analysis confirms this down-regulation of GABA_A_ receptor subunits (β3 and γ1) in DRG neurons induced by chronic morphine treatment. The functional consequence of this decreased GABA_A_ receptor synthesis in DRG neurons could be a reduction in the spinal pre-synaptic GABAergic tone, ultimately resulting in a persistent alteration of synaptic signalling.

### DNA microarray analysis as a tool to dissect molecular mechanisms involved in opioid treatment

Our DNA microarray experiment was designed to address distinct yet complementary questions. First, which genes are differentially expressed by vincristine treatment at the end of the chronic analgesic treatment? This analysis is important to comprehending the mechanisms underlying the maintenance of neuropathic pain symptoms associated with vincristine treatment. Since our DNA microarray experiment was performed days after the end of the treatment and several weeks after the development of neuropathic pain [17], [28], these genes are likely to be involved in the maintenance (and not development) of neuropathic pain symptoms. Second, which genes are differentially expressed in control animals when they are chronically treated with oxycodone or morphine? As stated above, chronic morphine treatment leads to an attenuation of its analgesic effect and the development of tolerance. Understanding the different targets of gene alteration induced by morphine at a steady state is important to clarifying the molecular mechanisms involved in the drug action and the development of this tolerance. As a final point, analysing the genes differentially expressed by oxycodone or morphine treatment in vincristine-treated animals provides us with further insight into the molecular mechanisms controlling the sustained analgesic effect of opioids in the neuropathic pain state.

Although these points are all important to address, their thorough pursuit was beyond the scope of this study. Future studies that aim to inquire into these different questions should greatly benefit from the complete database of dysregulated genes that we report here.

### The GABA_B_ receptor is involved in the long-lasting analgesic effect of oxycodone

The spinal cord is the site of both pro- and anti-nociceptive modulations. Released from primary afferent fibres, Substance P (SP), CGRP and glutamate exacerbate neuronal activity and have a pro-nociceptive action. Conversely, activation of GABA_B_ receptors inhibits voltage-dependent calcium channels via G-protein-coupled adenylate cyclase [42], [43] and reduces the release of excitatory amino acids and SP from primary afferent terminals in the spinal cord [44], [45]. Therefore, an increase in the GABAergic tonus decreases neuronal excitation and has an anti-nociceptive property.

In vincristine-treated animals (but not in naive animals), chronic treatment with oxycodone specifically induced an up-regulation of GABA_B2_ receptor expression as compared to vincristine-morphine treated rats. We also found that GABA_B2_ receptors were expressed in superficial layers of the dorsal horn, particularly in glutamatergic presynaptic terminals containing CGRP. As previously reported, the up-regulation of GABA_B_ receptors in DRG neurons leads to an increase in inhibitory transmission tonus and a decrease in excitatory transmission tonus, which could explain the long-lasting analgesic effect observed during chronic oxycodone administration in neuropathic pain conditions. Our hypothesis is that the up-regulation of GABA_B2_ receptor expression in CGRP sensory afferents observed after chronic oxycodone treatment may counteract the increased neuronal excitability induced by vincristine treatment. The inhibition of oxycodone's analgesic effect following an intrathecal injection of a selective GABAB receptor antagonist confirms this hypothesis. Therefore, the alleviating effect of oxycodone in neuropathic pain condition seems to involve the spinal GABAergic tonus though GABAB receptors.

Although functional GABA_B_ receptors must be comprised of a heterodimer of GABA_B1_ and GABA_B2_ receptor subtypes to be active [31], some studies report tissues in which the expression of GABA_B1_ and GABA_B2_ receptors do not overlap. For example, some tissues only express GABA_B1_ receptor subtypes (and not GABA_B2_), suggesting a distinct role for GABA_B1_ in the absence of GABA_B2_ [25]. In addition, independent maturation of GABA_B1_ and GABA_B2_ receptor subtypes has been observed during postnatal development in the brain [46]. It has been demonstrated that no biochemical or functional responses from the GABA_B_ receptor (or homomeric GBR1 or GBR2) are detectable in the brain or retina of a GABA_B1_ -/- mouse mutant. [47]–[51]. There is a partial preservation of the GABA_B2_ receptor subtype staining in the brain of GABA_B1_ -/- juvenile mice, suggesting a functional role for GABA_B2_ receptor subtype proteins in the immature brain, in addition to its contribution as a dimer in GABA_B_ receptor complexes[46]. Together, these data suggest that the GABA_B1_ and GABA_B2_ receptor subtypes may play other roles, independent of the presence of their partner. We could not exclude a different role for the up-regulation of the GABA_B2_ receptor subtype in our model.

In conclusion, this study provides evidence that treatment with oxycodone produces a profound anti-nociceptive effect under a neuropathic pain-like state. Furthermore, our results reveal a thorough list of genes that are dysregulated in DRGs at the end of chronic analgesic treatment. These findings will be indispensable to future studies that aim to understand the analgesic effects of oxycodone as compared to morphine in chemotherapy-induced neuropathic pain.

## Supporting Information

Figure S1 **Anti-GABA_B2_ receptor antibody specificity.** A: Representative western blotting of one spinal cord lysate, using the anti-GABA_B2_ receptor antibody. A single band was observed at 106–110 kDa, corresponding to the expected weight of the GABA_B2_ receptor protein. B: Anti-GABA_B2_ immunostaining on a spinal cord section. Representative example of GABA_B2_ staining in the dorsal horn, located primarily in the superficial laminae (arrowheads). Some staining was observed in deep laminae as well (arrows). Spinal cord sections incubated without primary antibody showed no staining (data not shown).
(TIF)

Figure S2 **Time-course of dynamic mechanical sensitivity.** At D15, rats in all vincristine-treated groups suffered from dynamic mechanical allodynia (A) and hyperesthesia (B). A: Mean number of positive responses to the smooth paint-brush test. The measured dynamic mechanical allodynia was totally reversed after a single injection of morphine or oxycodone, although only oxycodone maintained an analgesic effect at the end of the chronic analgesic treatment (D19). B: Mean number of positive responses to the rough paint-brush test. Only oxycodone maintained an analgesic effect at the end of the chronic analgesic treatment (D19). All data are expressed as mean ± SEM. (n = 7 vincristine-morphine treated rats; n = 6 saline-morphine treated rats), (n = 7 vincristine-oxycodone treated rats; n = 6 saline-oxycodone treated rats), and (n = 7 vincristine-saline treated rats; n = 6 saline-saline treated rats). *p<0.05: vincristine-oxycodone treated rats vs. vincristine-morphine treated rats; † p<0.05, †† p<0.01: vincristine-oxycodone treated rats vs. vincristine-saline treated rats; § p<0.05: vincristine-morphine treated rats vs. vincristine-saline treated rats. For figure clarity, the statistical significance symbols for the vincristine vs. saline groups are not shown.
(TIF)

Figure S3 **Colocalisation between GABA_B1_ and GABA_B2_ receptors in sensory neurons.** **A–F**: Double immunostaining showing the colocalisation of GABA_B1_ receptor (A, D) with GABA_B2_ receptor (D, E) in the DRG (A–C) and in the spinal dorsal horn (D–F) of a representative vincristine-oxycodone treated animal. C and F are merged images of A and B, and D and E, respectively. The results indicate colocalisation of both GABA_B1 and_ GABA_B2_ in small DRG neurons (arrow in A, B and C) and in the superficial spinal dorsal horn (arrowheads in the magnification of D, E and F). A–C, scale bar  = 30 μm; D–F, scale bar  = 150 μm.
(TIF)

Figure S4 **The GABA_B2_ receptor and CGRP colocalise in superficial laminae of the dorsal horn.** A–C: Double immunostaining showing CGRP (A, C, red)/GABA_B2_ receptor (B, C, green) positive fibres in a vincristine-oxycodone treated animal. C is a merged image of A and B, showing colocalisation in laminae I and II. D–F: Double immunostaining of IB4 (D, F, green)/GABA_B2_ receptor (E, F, red) indicates little colocalisation in a vincristine-oxycodone treated animal. Colocalisation was observed principally in laminae II ‘out’. F is a merge of D and E. Scale bar  = 100 μm.
(TIF)

Figure S5 **The acute analgesic effect of oxycodone is not reversed by i.t. injection of a GABA_B_ antagonist.** A, B: Effect of intrathecal saclofen (10 μg) injection on the acute analgesic effect of oxycodone on static mechanical allodynia (A) and static mechanical hyperalgesia (B). After i.t. injection of saclofen, the analgesic effect of a single injection of oxycodone at D15 was not modified (ns: vincristine-oxycodone-saclofen treated rats vs. vincristine-oxycodone-saline treated rats). All data are expressed as mean ± SEM. ††† p<0.001: vincristine-oxycodone-saclofen treated rats vs. vincristine-saline-saclofen treated rats; ### p<0.001: vincristine-oxycodone-saline treated rats vs. vincristine-saline-saline treated rats.
(TIF)

Figure S6 **The analgesic effect of morphine is not modified by i.t. injection of a GABA _B_ antagonist.** A, B: Effect of intrathecal saclofen (10 μg) injection on the acute (D15) and chronic (D19) analgesic effect of morphine on static mechanical allodynia (A) and static mechanical hyperalgesia (B). After i.t. injection of saclofen, the analgesic effect of a single injection of morphine at D15 was not modified (ns: vincristine-morphine-saclofen treated rats vs. vincristine-morphine-saline treated rats). The analgesic effect of morphine was decreased at D19 as compared to D15 (as expected), and was not modified by the i.t. injection of saclofen (ns). All data are expressed as mean ± SEM. § p<0.05, §§§ p<0.001: vincristine-morphine-saclofen treated rats vs. vincristine-saline-saclofen treated rats; # p<0.05, ### p<0.001: vincristine-morphine-saline treated rats vs. vincristine-saline-saline treated rats.
(TIF)

Table S1 **Complete list of genes dysregulated at the end of analgesic treatment.** Data are illustrated as follows: 1) Gene accession number; 2) Gene symbol; 3) Mean expression of the genes for the six different groups; 4) ident: an identifying z-z-z for the statistical analysis significance (z corresponding to the three tests; z = 1 if the null hypothesis is rejected with a risk of 5% or z = 0); 5) results of variance analysis between vincristine and control: p-value; 6) results of Student's t test between morphine and oxycodone in vincristine-treated rats: p-value and expression difference (morphine – oxycodone); 7) results of Student's t test between morphine and oxycodone in control rats: p-value and expression difference (morphine – oxycodone). Peach highlight corresponds to an identifying number of 1_1_1 *i.e*. with a statistically significant difference between vincristine and control, and morphine and oxycodone in vincristine-treated rats, and between morphine and oxycodone in control rats. Yellow highlight corresponds to an identifying number of 1_0_1 *i.e*. with a statistically significant difference between vincristine and control, and between morphine and oxycodone in control rats. Green highlight corresponds to an identifying number of 1_0_0 *i.e*. with a statistically significant difference between vincristine and control, only. Purple highlight corresponds to an identifying number of 0_1_1 *i.e*. with a statistically significant difference between morphine and oxycodone in vincristine-treated rats, and between morphine and oxycodone in control rats. Blue highlight corresponds to an identifying number of 0_1_0 *i.e*. with a statistically significant difference between morphine and oxycodone in vincristine-treated rats, only. In this group, there was significant differential expression of several genes corresponding to the GABA_A_ R subunit β3 (Gabrb3), the GABA_A_ R subunit γ1 (Gabrg1), and the GABA_B2_ receptor (Gabbr2). Dark blue highlight corresponds to an identifying number of 0_0_1 *i.e*. with a statistically significant difference between morphine and oxycodone in control rats, only.
(DOC)
