# Supplementary figures and images for: Molecular Mechanisms Underlying the Enhanced Analgesic Effect of Oxycodone Compared to Morphine in Chemotherapy-Induced Neuropathic Pain
Source: PLoS One. 2014 Mar 11;9(3):e91297. doi: 10.1371/journal.pone.0091297 (PMC3949760; doi:10.1371/journal.pone.0091297)

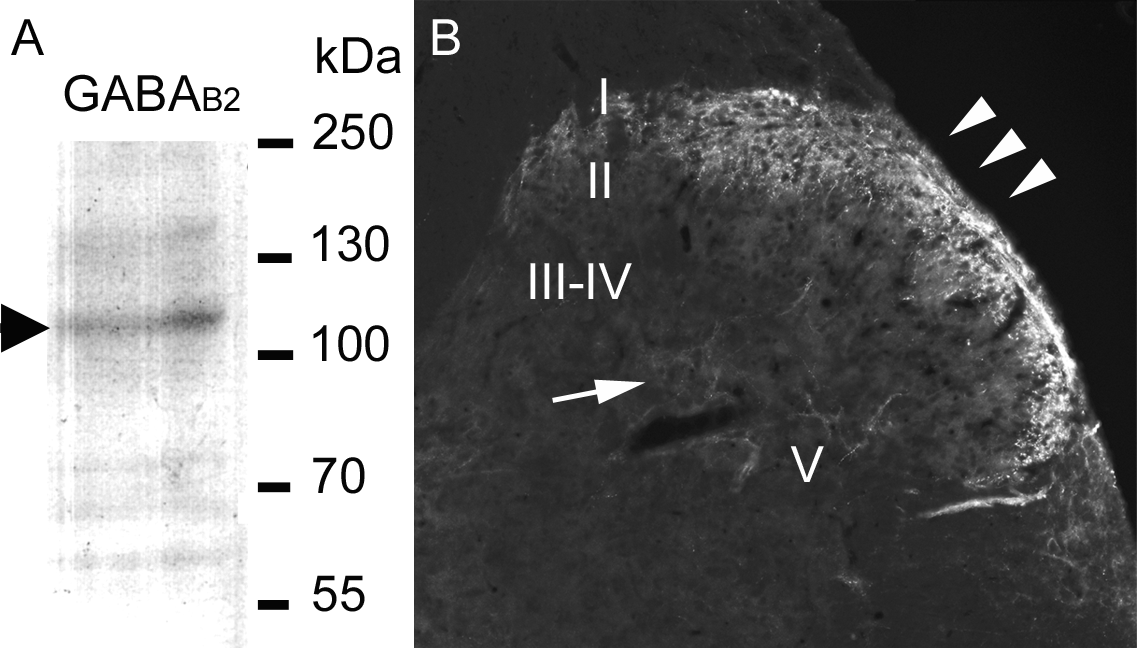

Supplement: Figure S1 — Anti-GABAB2 receptor antibody specificity. A: Representative western blotting of one spinal cord lysate, using the anti-GABAB2 receptor antibody. A single band was observed at 106–110 kDa, corresponding to the expected weight of the GABAB2 receptor protein. B: Anti-GABAB2 immunostaining on a spinal cord section. Representative example of GABAB2 staining in the dorsal horn, located primarily in the superficial laminae (arrowheads). Some staining was observed in deep laminae as well (arrows). Spinal cord sections incubated without primary antibody showed no staining (data not shown). (TIF) [file pone.0091297.s001.tif]

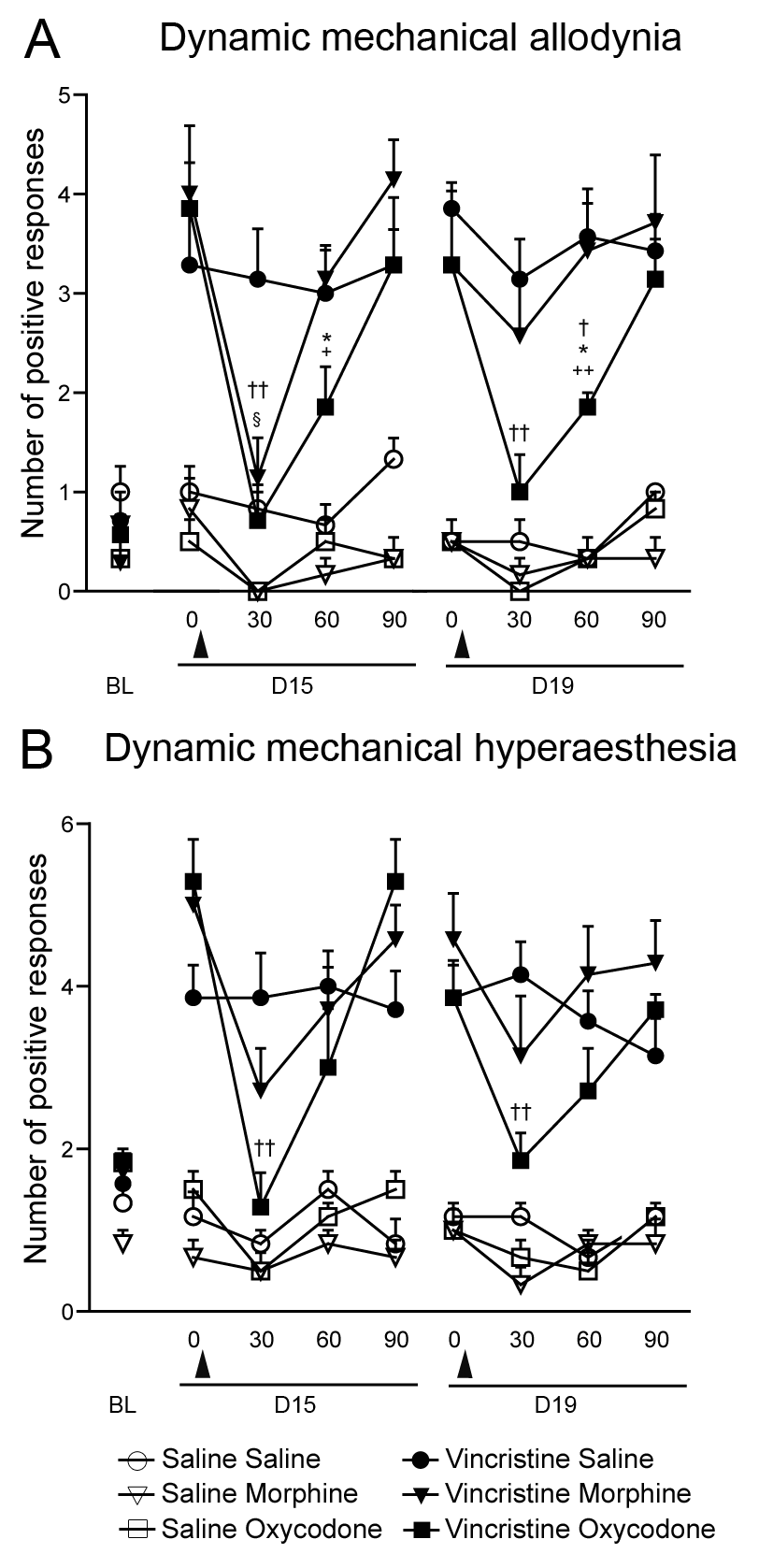

Supplement: Figure S2 — Time-course of dynamic mechanical sensitivity. At D15, rats in all vincristine-treated groups suffered from dynamic mechanical allodynia (A) and hyperesthesia (B). A: Mean number of positive responses to the smooth paint-brush test. The measured dynamic mechanical allodynia was totally reversed after a single injection of morphine or oxycodone, although only oxycodone maintained an analgesic effect at the end of the chronic analgesic treatment (D19). B: Mean number of positive responses to the rough paint-brush test. Only oxycodone maintained an analgesic effect at the end of the chronic analgesic treatment (D19). All data are expressed as mean ± SEM. (n = 7 vincristine-morphine treated rats; n = 6 saline-morphine treated rats), (n = 7 vincristine-oxycodone treated rats; n = 6 saline-oxycodone treated rats), and (n = 7 vincristine-saline treated rats; n = 6 saline-saline treated rats). *p<0.05: vincristine-oxycodone treated rats vs. vincristine-morphine treated rats; † p<0.05, †† p<0.01: vincristine-oxycodone treated rats vs. vincristine-saline treated rats; § p<0.05: vincristine-morphine treated rats vs. vincristine-saline treated rats. For figure clarity, the statistical significance symbols for the vincristine vs. saline groups are not shown. (TIF) [file pone.0091297.s002.tif]

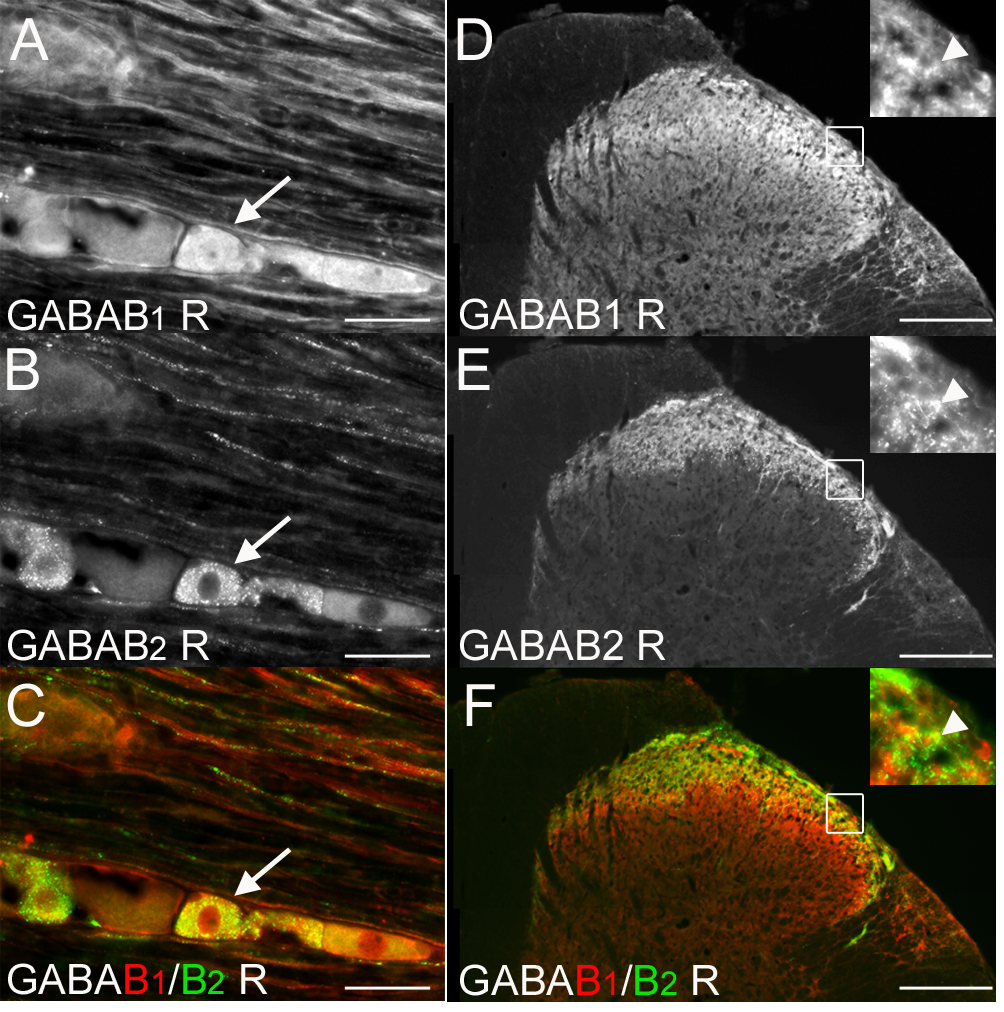

Supplement: Figure S3 — Colocalisation between GABAB1 and GABAB2 receptors in sensory neurons. A–F: Double immunostaining showing the colocalisation of GABAB1 receptor (A, D) with GABAB2 receptor (D, E) in the DRG (A–C) and in the spinal dorsal horn (D–F) of a representative vincristine-oxycodone treated animal. C and F are merged images of A and B, and D and E, respectively. The results indicate colocalisation of both GABAB1 and GABAB2 in small DRG neurons (arrow in A, B and C) and in the superficial spinal dorsal horn (arrowheads in the magnification of D, E and F). A–C, scale bar = 30 μm; D–F, scale bar = 150 μm. (TIF) [file pone.0091297.s003.tif]

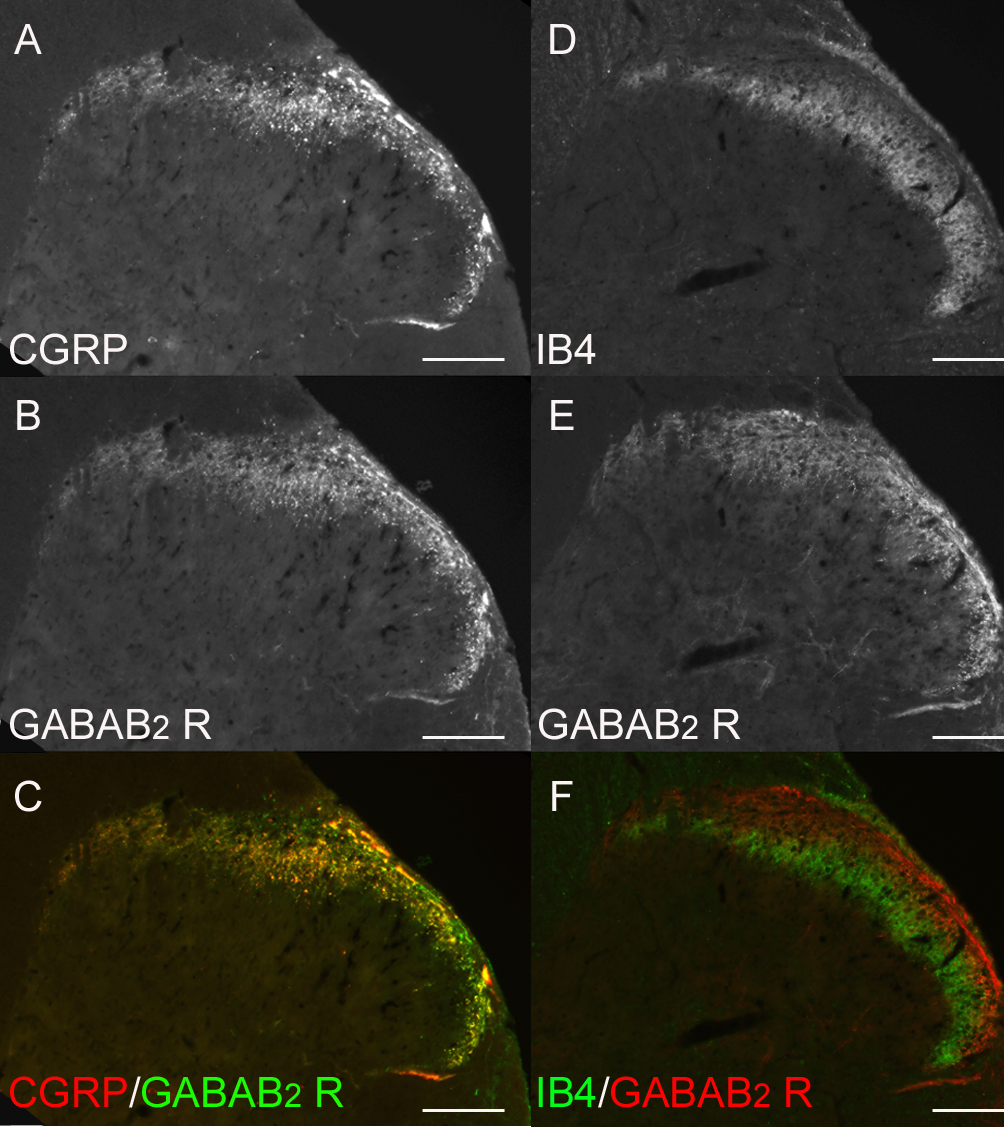

Supplement: Figure S4 — The GABAB2 receptor and CGRP colocalise in superficial laminae of the dorsal horn. A–C: Double immunostaining showing CGRP (A, C, red)/GABAB2 receptor (B, C, green) positive fibres in a vincristine-oxycodone treated animal. C is a merged image of A and B, showing colocalisation in laminae I and II. D–F: Double immunostaining of IB4 (D, F, green)/GABAB2 receptor (E, F, red) indicates little colocalisation in a vincristine-oxycodone treated animal. Colocalisation was observed principally in laminae II ‘out’. F is a merge of D and E. Scale bar = 100 μm. (TIF) [file pone.0091297.s004.tif]

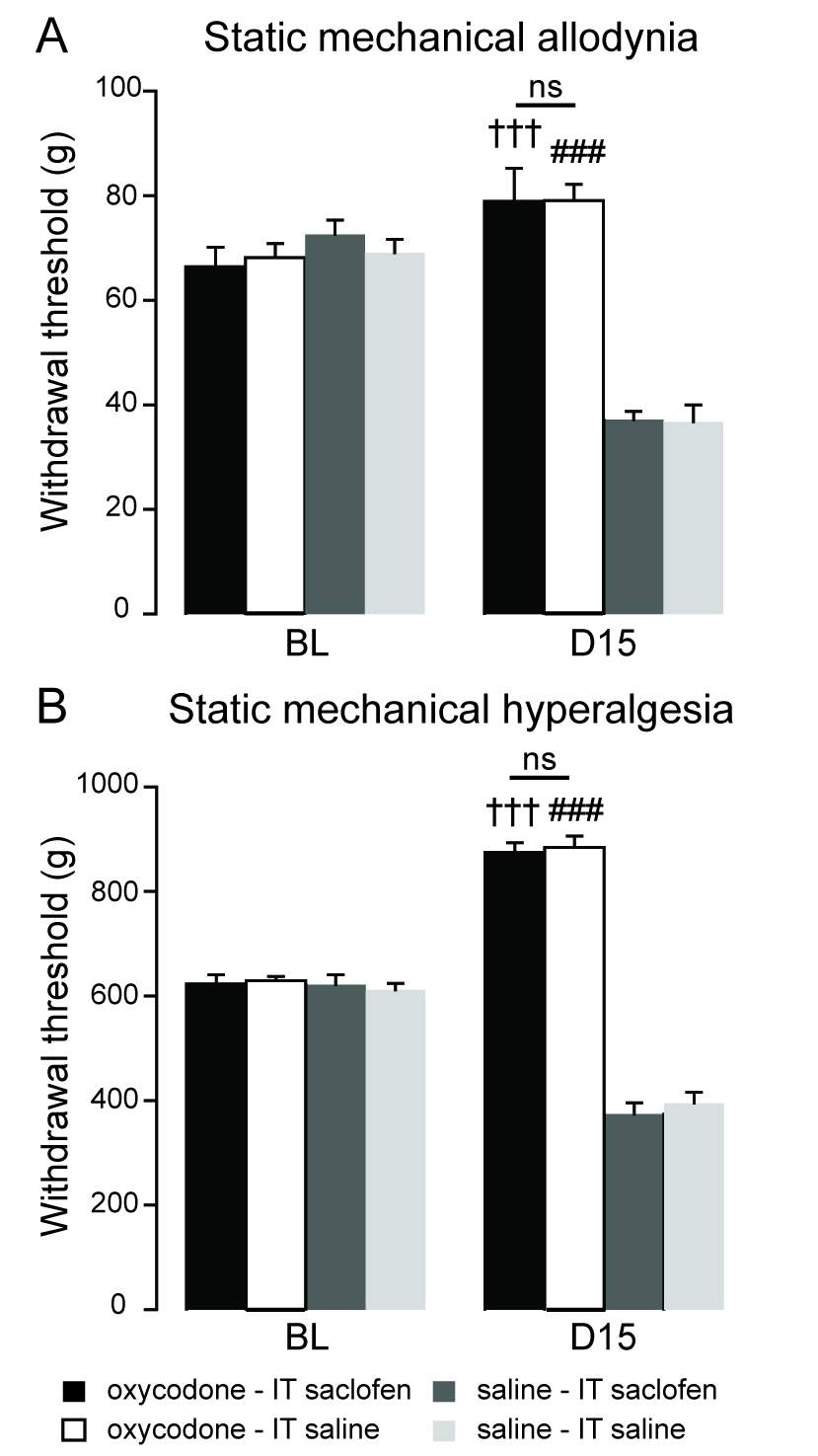

Supplement: Figure S5 — The acute analgesic effect of oxycodone is not reversed by i.t. injection of a GABAB antagonist. A, B: Effect of intrathecal saclofen (10 μg) injection on the acute analgesic effect of oxycodone on static mechanical allodynia (A) and static mechanical hyperalgesia (B). After i.t. injection of saclofen, the analgesic effect of a single injection of oxycodone at D15 was not modified (ns: vincristine-oxycodone-saclofen treated rats vs. vincristine-oxycodone-saline treated rats). All data are expressed as mean ± SEM. ††† p<0.001: vincristine-oxycodone-saclofen treated rats vs. vincristine-saline-saclofen treated rats; ### p<0.001: vincristine-oxycodone-saline treated rats vs. vincristine-saline-saline treated rats. (TIF) [file pone.0091297.s005.tif]

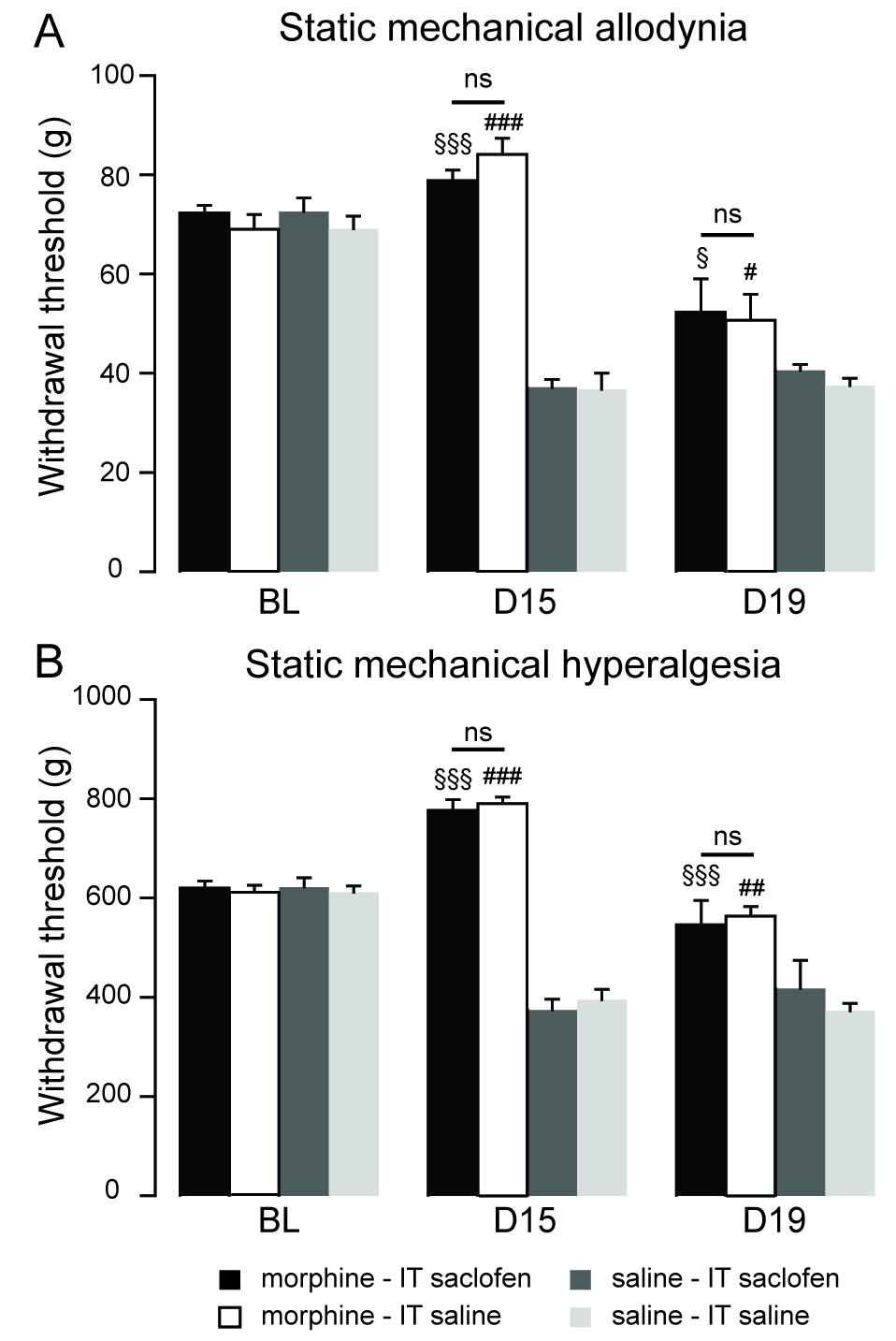

Supplement: Figure S6 — The analgesic effect of morphine is not modified by i.t. injection of a GABA B antagonist. A, B: Effect of intrathecal saclofen (10 μg) injection on the acute (D15) and chronic (D19) analgesic effect of morphine on static mechanical allodynia (A) and static mechanical hyperalgesia (B). After i.t. injection of saclofen, the analgesic effect of a single injection of morphine at D15 was not modified (ns: vincristine-morphine-saclofen treated rats vs. vincristine-morphine-saline treated rats). The analgesic effect of morphine was decreased at D19 as compared to D15 (as expected), and was not modified by the i.t. injection of saclofen (ns). All data are expressed as mean ± SEM. § p<0.05, §§§ p<0.001: vincristine-morphine-saclofen treated rats vs. vincristine-saline-saclofen treated rats; # p<0.05, ### p<0.001: vincristine-morphine-saline treated rats vs. vincristine-saline-saline treated rats. (TIF) [file pone.0091297.s006.tif]
