# Supplementary material for: Molecular Mechanisms Underlying the Enhanced Analgesic Effect of Oxycodone Compared to Morphine in Chemotherapy-Induced Neuropathic Pain
Source: PLoS One. 2014 Mar 11;9(3):e91297. doi: 10.1371/journal.pone.0091297 (PMC3949760; doi:10.1371/journal.pone.0091297)
Supplement: Table S1 — Complete list of genes dysregulated at the end of analgesic treatment. Data are illustrated as follows: 1) Gene accession number; 2) Gene symbol; 3) Mean expression of the genes for the six different groups; 4) ident: an identifying z-z-z for the statistical analysis significance (z corresponding to the three tests; z = 1 if the null hypothesis is rejected with a risk of 5% or z = 0); 5) results of variance analysis between vincristine and control: p-value; 6) results of Student's t test between morphine and oxycodone in vincristine-treated rats: p-value and expression difference (morphine – oxycodone); 7) results of Student's t test between morphine and oxycodone in control rats: p-value and expression difference (morphine – oxycodone). Peach highlight corresponds to an identifying number of 1_1_1 i.e. with a statistically significant difference between vincristine and control, and morphine and oxycodone in vincristine-treated rats, and between morphine and oxycodone in control rats. Yellow highlight corresponds to an identifying number of 1_0_1 i.e. with a statistically significant difference between vincristine and control, and between morphine and oxycodone in control rats. Green highlight corresponds to an identifying number of 1_0_0 i.e. with a statistically significant difference between vincristine and control, only. Purple highlight corresponds to an identifying number of 0_1_1 i.e. with a statistically significant difference between morphine and oxycodone in vincristine-treated rats, and between morphine and oxycodone in control rats. Blue highlight corresponds to an identifying number of 0_1_0 i.e. with a statistically significant difference between morphine and oxycodone in vincristine-treated rats, only. In this group, there was significant differential expression of several genes corresponding to the GABAA R subunit β3 (Gabrb3), the GABAA R subunit γ1 (Gabrg1), and the GABAB2 receptor (Gabbr2). Dark blue highlight corresponds to an identifying number o [file pone.0091297.s007.doc]

|  |  |  |  |  | |  |  |  | | Variance analysis | | Mor vs Oxy | | | Mor vs Oxy | | |
| --- | --- | --- | --- | --- | --- | --- | --- | --- | --- | --- | --- | --- | --- | --- | --- | --- | --- |
|  |  |  |  |  |  | |  |  | | Vin vs Control | | Vincritsine animals | | | Control animals | | |
| ACCESSION | SYMBOL | V-Mor | V-Oxy | V-NaCl | S-Mor | | S-Oxy | S-NaCl | ident | p | p | | difference | p | | difference |  |
| NM_171993 | Cdc20 | 6,43 | 6,32 | 6,35 | 6,38 | | 6,55 | 6,44 | 1_1_1 | 0,01 | 0,04 | | 0,11 | 0,01 | | -0,17 |  |
| NM_031010 | Alox15 | 10,66 | 9,43 | 10,22 | 6,43 | | 6,29 | 6,38 | 1_1_0 | 0,00 | 0,01 | | 1,22 | 0,74 | | 0,13 |  |
| NM_021657 | Phlpp | 8,19 | 8,07 | 8,09 | 8,21 | | 8,21 | 8,20 | 1_1_0 | 0,01 | 0,03 | | 0,12 | 0,96 | | 0,00 |  |
| NM_001004132 | Pctk1 | 8,50 | 8,71 | 8,54 | 8,84 | | 8,73 | 8,70 | 1_1_0 | 0,01 | 0,04 | | -0,21 | 0,28 | | 0,10 |  |
| NM_144737 | Fmo2 | 7,19 | 7,67 | 7,27 | 7,01 | | 7,03 | 7,01 | 1_1_0 | 0,01 | 0,04 | | -0,48 | 0,92 | | -0,02 |  |
| XM_220167 | Coro7 | 10,43 | 10,37 | 10,46 | 10,42 | | 10,52 | 10,47 | 1_0_1 | 0,04 | 0,13 | | 0,06 | 0,02 | | -0,10 |  |
| NM_178101 | Plod3 | 9,72 | 9,66 | 9,64 | 9,70 | | 9,81 | 9,71 | 1_0_1 | 0,04 | 0,27 | | 0,06 | 0,03 | | -0,11 |  |
| NM_031700 | Cldn3 | 6,91 | 6,83 | 6,79 | 6,60 | | 6,83 | 6,76 | 1_0_1 | 0,04 | 0,37 | | 0,08 | 0,02 | | -0,23 |  |
| NM_013190 | Pfkl | 8,01 | 8,06 | 7,97 | 7,99 | | 7,79 | 7,97 | 1_0_1 | 0,03 | 0,51 | | -0,05 | 0,02 | | 0,20 |  |
| NM_022600 | Adcy5 | 6,44 | 6,48 | 6,40 | 6,52 | | 6,70 | 6,66 | 1_0_1 | 0,00 | 0,60 | | -0,04 | 0,03 | | -0,18 |  |
| NM_012498 | Akr1b4 | 12,97 | 13,00 | 12,98 | 13,15 | | 12,97 | 13,14 | 1_0_1 | 0,02 | 0,68 | | -0,03 | 0,01 | | 0,18 |  |
| NM_181382 | Colm | 9,05 | 9,03 | 8,94 | 9,30 | | 9,08 | 9,28 | 1_0_1 | 0,00 | 0,82 | | 0,02 | 0,04 | | 0,22 |  |
| XM_215451 | Cspg2 | 6,77 | 6,77 | 6,93 | 6,75 | | 6,54 | 6,76 | 1_0_1 | 0,02 | 0,97 | | 0,00 | 0,04 | | 0,21 |  |
| NM_030862 | Mlp | 8,05 | 8,06 | 8,07 | 8,30 | | 8,06 | 8,21 | 1_0_1 | 0,04 | 0,97 | | 0,00 | 0,03 | | 0,24 |  |
| NM_012976 | Lgals5 | 7,48 | 6,76 | 6,54 | 6,17 | | 6,18 | 6,20 | 1_0_0 | 0,00 | 0,06 | | 0,72 | NaN | | NaN |  |
| NM_133513 | Muc10 | 6,22 | 6,04 | 6,07 | 5,98 | | 6,02 | 5,97 | 1_0_0 | 0,03 | 0,06 | | 0,17 | 0,65 | | -0,04 |  |
| XM_579405 | Hmgcs1 | 13,60 | 13,48 | 13,51 | 13,57 | | 13,64 | 13,65 | 1_0_0 | 0,02 | 0,06 | | 0,11 | 0,25 | | -0,07 |  |
| NM_030830 | Luzp1 | 9,44 | 9,65 | 9,52 | 10,04 | | 10,20 | 10,01 | 1_0_0 | 0,00 | 0,07 | | -0,21 | 0,16 | | -0,16 |  |
| NM_199114 | Fgfrl1 | 6,92 | 6,76 | 6,81 | 6,97 | | 6,97 | 6,89 | 1_0_0 | 0,04 | 0,08 | | 0,17 | 0,96 | | 0,00 |  |
| NM_031776 | Gda | 11,28 | 11,46 | 11,34 | 11,20 | | 11,12 | 11,39 | 1_0_0 | 0,03 | 0,08 | | -0,18 | 0,41 | | 0,08 |  |
| NM_133294 | Hemgn | 7,15 | 6,76 | 6,82 | 6,13 | | 6,18 | 6,26 | 1_0_0 | 0,00 | 0,09 | | 0,39 | NaN | | NaN |  |
| NM_017138 | Lamr1 | 12,79 | 12,71 | 12,81 | 12,68 | | 12,76 | 12,68 | 1_0_0 | 0,04 | 0,10 | | 0,08 | 0,11 | | -0,08 |  |
| XM_343040 | Rrm2 | 6,35 | 6,10 | 6,28 | 6,04 | | 5,96 | 6,03 | 1_0_0 | 0,02 | 0,11 | | 0,25 | NaN | | NaN |  |
| NM_019904 | Lgals1 | 13,62 | 13,73 | 13,63 | 13,77 | | 13,73 | 13,74 | 1_0_0 | 0,04 | 0,12 | | -0,11 | 0,60 | | 0,04 |  |
| NM_147146 | sarip | 9,59 | 9,75 | 9,69 | 9,96 | | 9,87 | 9,93 | 1_0_0 | 0,00 | 0,12 | | -0,17 | 0,40 | | 0,09 |  |
| NM_145677 | Slc25a25 | 9,76 | 9,68 | 9,70 | 9,83 | | 9,76 | 9,74 | 1_0_0 | 0,03 | 0,12 | | 0,08 | 0,16 | | 0,07 |  |
| NM_019194 | Tef | 6,65 | 6,80 | 6,86 | 6,43 | | 6,54 | 6,79 | 1_0_0 | 0,00 | 0,12 | | -0,15 | 0,28 | | -0,11 |  |
| NM_001006958 | Sdad1 | 6,05 | 6,16 | 6,11 | 6,21 | | 6,23 | 6,15 | 1_0_0 | 0,04 | 0,12 | | -0,11 | NaN | | NaN |  |
| NM_173118 | Npc2 | 12,11 | 12,22 | 12,19 | 12,35 | | 12,21 | 12,24 | 1_0_0 | 0,04 | 0,13 | | -0,11 | 0,05 | | 0,15 |  |
| NM_012491 | Add2 | 6,90 | 6,68 | 6,76 | 6,22 | | 6,28 | 6,33 | 1_0_0 | 0,00 | 0,13 | | 0,22 | 0,69 | | -0,06 |  |
| NM_031678 | Per2 | 8,23 | 8,64 | 8,25 | 8,06 | | 7,98 | 7,94 | 1_0_0 | 0,02 | 0,13 | | -0,41 | 0,78 | | 0,08 |  |
| NM_001009953 | Rtn3 | 6,64 | 6,55 | 6,59 | 6,70 | | 6,70 | 6,62 | 1_0_0 | 0,04 | 0,14 | | 0,09 | 0,99 | | 0,00 |  |
| NM_173140 | Cryba2 | 7,05 | 7,22 | 7,06 | 7,16 | | 7,35 | 7,40 | 1_0_0 | 0,01 | 0,14 | | -0,17 | 0,10 | | -0,19 |  |
| XM_579743 | Tcf19 | 6,28 | 6,36 | 6,26 | 6,23 | | 6,22 | 6,25 | 1_0_0 | 0,04 | 0,16 | | -0,08 | 0,87 | | 0,01 |  |
| NM_001008277 | Tbl3 | 8,25 | 8,19 | 8,22 | 8,33 | | 8,25 | 8,29 | 1_0_0 | 0,02 | 0,16 | | 0,07 | 0,11 | | 0,08 |  |
| XM_341133 | Lamc1 | 6,74 | 6,93 | 6,80 | 7,24 | | 7,23 | 7,20 | 1_0_0 | 0,00 | 0,17 | | -0,19 | 0,94 | | 0,01 |  |
| NM_022392 | Insig1 | 9,90 | 9,79 | 9,93 | 10,02 | | 9,95 | 10,07 | 1_0_0 | 0,00 | 0,18 | | 0,10 | 0,31 | | 0,08 |  |
| XM_236956 | Enpp5 | 8,25 | 8,12 | 8,15 | 8,11 | | 7,95 | 8,09 | 1_0_0 | 0,04 | 0,18 | | 0,13 | 0,12 | | 0,15 |  |
| XM_340822 | Per1 | 8,13 | 8,53 | 8,22 | 7,95 | | 7,66 | 7,80 | 1_0_0 | 0,01 | 0,19 | | -0,40 | 0,34 | | 0,29 |  |
| NM_012752 | Cd24 | 11,24 | 11,37 | 11,23 | 11,10 | | 11,19 | 11,18 | 1_0_0 | 0,04 | 0,19 | | -0,13 | 0,35 | | -0,09 |  |
| NM_198769 | Tlr2 | 6,82 | 6,66 | 6,89 | 6,89 | | 6,96 | 6,99 | 1_0_0 | 0,04 | 0,20 | | 0,16 | 0,61 | | -0,06 |  |
| NM_024356 | Gch | 6,84 | 6,53 | 6,69 | 6,12 | | 6,33 | 6,25 | 1_0_0 | 0,00 | 0,21 | | 0,31 | 0,38 | | -0,21 |  |
| NM_030853 | Lat | 6,61 | 6,49 | 6,52 | 6,42 | | 6,40 | 6,43 | 1_0_0 | 0,03 | 0,22 | | 0,12 | 0,83 | | 0,02 |  |
| NM_019357 | Vil2 | 8,00 | 7,90 | 7,95 | 8,12 | | 8,04 | 8,03 | 1_0_0 | 0,03 | 0,23 | | 0,10 | 0,36 | | 0,08 |  |
| XM_340809 | Rasd1 | 6,91 | 6,81 | 6,90 | 6,73 | | 6,82 | 6,76 | 1_0_0 | 0,05 | 0,23 | | 0,10 | 0,27 | | -0,09 |  |
| XM_579342 | Tacr1 | 6,21 | 6,13 | 6,11 | 6,08 | | 6,03 | 6,03 | 1_0_0 | 0,03 | 0,24 | | 0,08 | NaN | | NaN |  |
| NM_031753 | Alcam | 11,37 | 11,24 | 11,25 | 11,08 | | 11,14 | 11,26 | 1_0_0 | 0,05 | 0,24 | | 0,12 | 0,53 | | -0,07 |  |
| NM_019386 | Tgm2 | 6,36 | 6,42 | 6,37 | 6,46 | | 6,51 | 6,47 | 1_0_0 | 0,01 | 0,26 | | -0,06 | 0,37 | | -0,05 |  |
| NM_133316 | Mutyh | 6,14 | 6,21 | 6,29 | 6,17 | | 6,03 | 6,09 | 1_0_0 | 0,00 | 0,27 | | -0,07 | NaN | | NaN |  |
| NM_134407 | Akr7a2 | 7,09 | 7,00 | 7,06 | 7,14 | | 7,25 | 7,07 | 1_0_0 | 0,05 | 0,28 | | 0,09 | 0,22 | | -0,10 |  |
| NM_134455 | Cx3cl1 | 8,20 | 8,05 | 7,90 | 8,28 | | 8,47 | 7,97 | 1_0_0 | 0,03 | 0,29 | | 0,16 | 0,20 | | -0,19 |  |
| XM_579459 | Lgals7 | 6,33 | 6,27 | 6,34 | 6,37 | | 6,41 | 6,40 | 1_0_0 | 0,03 | 0,30 | | 0,06 | 0,57 | | -0,03 |  |
| NM_133416 | Bcl2a1 | 6,91 | 6,77 | 6,90 | 7,05 | | 7,16 | 7,01 | 1_0_0 | 0,01 | 0,30 | | 0,14 | 0,39 | | -0,12 |  |
| XM_234720 | Dnah11 | 6,20 | 6,28 | 6,35 | 6,40 | | 6,32 | 6,53 | 1_0_0 | 0,01 | 0,31 | | -0,09 | 0,35 | | 0,08 |  |
| NM_019187 | Coq3 | 9,54 | 9,66 | 9,56 | 9,77 | | 9,90 | 9,88 | 1_0_0 | 0,00 | 0,31 | | -0,13 | 0,31 | | -0,13 |  |
| NM_031979 | Csda | 10,40 | 10,22 | 10,33 | 10,04 | | 9,80 | 9,92 | 1_0_0 | 0,00 | 0,31 | | 0,18 | 0,20 | | 0,23 |  |
| NM_022285 | Hapln2 | 7,78 | 7,63 | 7,81 | 7,90 | | 8,02 | 8,17 | 1_0_0 | 0,00 | 0,32 | | 0,14 | 0,39 | | -0,12 |  |
| NM_012758 | Syk | 6,12 | 6,20 | 6,18 | 6,30 | | 6,23 | 6,30 | 1_0_0 | 0,05 | 0,32 | | -0,09 | 0,39 | | 0,08 |  |
| XM_579599 | Sec22l2 | 6,42 | 6,36 | 6,38 | 6,48 | | 6,47 | 6,46 | 1_0_0 | 0,04 | 0,32 | | 0,06 | 0,95 | | 0,00 |  |
| NM_031826 | Fbn2 | 7,08 | 6,99 | 7,20 | 7,24 | | 7,24 | 7,18 | 1_0_0 | 0,03 | 0,33 | | 0,09 | 0,94 | | 0,01 |  |
| NM_017307 | Slc25a1 | 10,97 | 10,88 | 10,95 | 10,99 | | 11,17 | 11,16 | 1_0_0 | 0,00 | 0,34 | | 0,09 | 0,07 | | -0,18 |  |
| NM_017151 | Rps15 | 11,42 | 11,54 | 11,48 | 11,62 | | 11,61 | 11,69 | 1_0_0 | 0,04 | 0,36 | | -0,12 | 0,93 | | 0,01 |  |
| NM_199504 | Pcdha2 | 6,32 | 6,39 | 6,32 | 6,32 | | 6,19 | 6,25 | 1_0_0 | 0,04 | 0,36 | | -0,06 | 0,08 | | 0,12 |  |
| XM_579564 | Gosr1 | 6,13 | 6,20 | 6,22 | 6,14 | | 6,04 | 6,08 | 1_0_0 | 0,04 | 0,36 | | -0,07 | 0,18 | | 0,11 |  |
| NM_053289 | Pap | 6,31 | 6,40 | 6,39 | 6,29 | | 6,18 | 6,17 | 1_0_0 | 0,02 | 0,38 | | -0,09 | NaN | | NaN |  |
| NM_001007729 | Pf4 | 9,50 | 8,98 | 9,93 | 8,41 | | 7,90 | 7,77 | 1_0_0 | 0,00 | 0,39 | | 0,53 | 0,41 | | 0,51 |  |
| NM_033234 | Hbb | 14,04 | 13,85 | 13,96 | 12,79 | | 12,71 | 13,03 | 1_0_0 | 0,00 | 0,40 | | 0,20 | 0,71 | | 0,08 |  |
| NM_053774 | Usp2 | 7,32 | 7,45 | 7,39 | 7,20 | | 7,18 | 7,19 | 1_0_0 | 0,05 | 0,40 | | -0,14 | 0,93 | | 0,01 |  |
| NM_139094 | Rbm16 | 9,67 | 9,72 | 9,86 | 9,62 | | 9,66 | 9,76 | 1_0_0 | 0,04 | 0,41 | | -0,05 | 0,47 | | -0,04 |  |
| NM_144762 | Baalc | 7,39 | 7,34 | 7,36 | 7,40 | | 7,48 | 7,44 | 1_0_0 | 0,03 | 0,42 | | 0,04 | 0,15 | | -0,08 |  |
| XM_579626 | Cda08 | 11,37 | 11,34 | 11,43 | 11,29 | | 11,29 | 11,39 | 1_0_0 | 0,05 | 0,43 | | 0,04 | 0,92 | | 0,00 |  |
| NM_001008510 | Isg20 | 6,38 | 6,30 | 6,26 | 6,18 | | 6,13 | 6,16 | 1_0_0 | 0,01 | 0,43 | | 0,07 | 0,55 | | 0,06 |  |
| NM_145089 | Asrgl1 | 10,92 | 10,87 | 10,98 | 11,03 | | 11,09 | 11,01 | 1_0_0 | 0,00 | 0,44 | | 0,05 | 0,30 | | -0,07 |  |
| NM_031530 | Ccl2 | 7,44 | 7,35 | 7,42 | 7,57 | | 7,53 | 7,64 | 1_0_0 | 0,02 | 0,44 | | 0,09 | 0,73 | | 0,04 |  |
| NM_012529 | Ckb | 11,66 | 11,61 | 11,61 | 11,74 | | 11,75 | 11,66 | 1_0_0 | 0,03 | 0,44 | | 0,05 | 0,84 | | -0,01 |  |
| NM_012656 | Sparc | 12,19 | 12,08 | 12,20 | 12,36 | | 12,32 | 12,41 | 1_0_0 | 0,02 | 0,45 | | 0,11 | 0,79 | | 0,04 |  |
| NM_130740 | Pacsin2 | 6,78 | 6,85 | 6,69 | 7,08 | | 7,09 | 7,04 | 1_0_0 | 0,00 | 0,48 | | -0,07 | 0,97 | | 0,00 |  |
| NM_022394 | Safb | 8,25 | 8,31 | 8,21 | 8,59 | | 8,59 | 8,58 | 1_0_0 | 0,00 | 0,50 | | -0,06 | 0,94 | | -0,01 |  |
| NM_138831 | Slc16a10 | 6,10 | 6,07 | 6,01 | 5,92 | | 5,89 | 5,91 | 1_0_0 | 0,00 | 0,52 | | 0,03 | NaN | | NaN |  |
| NM_053538 | Laptm5 | 9,24 | 9,18 | 9,26 | 9,36 | | 9,38 | 9,41 | 1_0_0 | 0,02 | 0,53 | | 0,07 | 0,85 | | -0,02 |  |
| NM_001009255 | Sfrs9 | 7,61 | 7,68 | 7,70 | 7,89 | | 7,92 | 7,85 | 1_0_0 | 0,00 | 0,55 | | -0,06 | 0,79 | | -0,03 |  |
| NM_052806 | Chrnb4 | 6,62 | 6,67 | 6,57 | 6,75 | | 6,75 | 6,68 | 1_0_0 | 0,04 | 0,55 | | -0,05 | 0,94 | | 0,01 |  |
| XM_579460 | Ctsb | 13,60 | 13,56 | 13,57 | 13,51 | | 13,47 | 13,50 | 1_0_0 | 0,02 | 0,56 | | 0,03 | 0,59 | | 0,03 |  |
| NM_133566 | Cst6 | 7,77 | 7,86 | 7,50 | 8,13 | | 7,95 | 8,13 | 1_0_0 | 0,00 | 0,56 | | -0,09 | 0,25 | | 0,18 |  |
| NM_199399 | Gpr108 | 7,62 | 7,69 | 7,64 | 7,76 | | 7,91 | 7,87 | 1_0_0 | 0,02 | 0,57 | | -0,08 | 0,27 | | -0,15 |  |
| NM_181087 | Cyp26b1 | 7,26 | 7,18 | 7,61 | 7,50 | | 7,64 | 7,40 | 1_0_0 | 0,05 | 0,57 | | 0,08 | 0,34 | | -0,13 |  |
| NM_031144 | Actb | 13,34 | 13,36 | 13,39 | 13,43 | | 13,43 | 13,42 | 1_0_0 | 0,04 | 0,58 | | -0,03 | 0,85 | | 0,01 |  |
| XM_212832 | Rpl10 | 12,68 | 12,64 | 12,73 | 12,61 | | 12,54 | 12,55 | 1_0_0 | 0,02 | 0,59 | | 0,04 | 0,41 | | 0,07 |  |
| NM_139081 | Oaz1 | 13,38 | 13,36 | 13,37 | 13,44 | | 13,44 | 13,41 | 1_0_0 | 0,03 | 0,59 | | 0,02 | 0,88 | | -0,01 |  |
| NM_031561 | Cd36 | 6,87 | 6,79 | 6,85 | 6,60 | | 6,61 | 6,73 | 1_0_0 | 0,03 | 0,60 | | 0,08 | 0,94 | | -0,01 |  |
| XM_579078 | Bace2 | 7,06 | 7,01 | 7,08 | 7,23 | | 7,18 | 7,22 | 1_0_0 | 0,01 | 0,60 | | 0,05 | 0,59 | | 0,05 |  |
| NM_017126 | Fdx1 | 9,08 | 9,04 | 9,05 | 9,20 | | 9,16 | 9,10 | 1_0_0 | 0,04 | 0,61 | | 0,04 | 0,54 | | 0,05 |  |
| NM_033237 | Gal | 7,07 | 7,15 | 7,02 | 6,77 | | 6,68 | 6,74 | 1_0_0 | 0,00 | 0,62 | | -0,08 | 0,58 | | 0,09 |  |
| NM_020306 | Adam17 | 8,94 | 8,88 | 8,92 | 9,10 | | 9,11 | 9,08 | 1_0_0 | 0,02 | 0,64 | | 0,06 | 0,97 | | 0,00 |  |
| NM_138882 | Pspla1 | 6,59 | 6,65 | 6,56 | 6,48 | | 6,40 | 6,44 | 1_0_0 | 0,04 | 0,64 | | -0,06 | 0,56 | | 0,08 |  |
| NM_173096 | Mx1 | 8,35 | 8,46 | 8,22 | 8,57 | | 8,65 | 8,94 | 1_0_0 | 0,01 | 0,65 | | -0,11 | 0,75 | | -0,08 |  |
| NM_145790 | Lipogenin | 6,21 | 6,17 | 6,15 | 6,06 | | 6,09 | 6,00 | 1_0_0 | 0,03 | 0,66 | | 0,04 | 0,78 | | -0,03 |  |
| NM_012614 | Npy | 8,65 | 8,37 | 8,85 | 7,14 | | 7,38 | 7,00 | 1_0_0 | 0,00 | 0,66 | | 0,28 | 0,71 | | -0,24 |  |
| NM_013106 | Gnai3 | 10,20 | 10,23 | 10,29 | 10,30 | | 10,31 | 10,32 | 1_0_0 | 0,04 | 0,67 | | -0,02 | 0,95 | | 0,00 |  |
| NM_017196 | Aif1 | 9,77 | 9,73 | 10,00 | 9,92 | | 10,01 | 10,03 | 1_0_0 | 0,02 | 0,67 | | 0,04 | 0,36 | | -0,10 |  |
| NM_145774 | Rab38 | 6,10 | 6,13 | 6,14 | 6,01 | | 6,11 | 6,00 | 1_0_0 | 0,01 | 0,67 | | -0,02 | 0,06 | | -0,10 |  |
| NM_053908 | Ptpn6 | 7,57 | 7,52 | 7,64 | 7,68 | | 7,74 | 7,73 | 1_0_0 | 0,05 | 0,67 | | 0,05 | 0,60 | | -0,06 |  |
| XM_579441 | Adam1a | 5,95 | 5,99 | 6,00 | 6,02 | | 6,16 | 6,12 | 1_0_0 | 0,03 | 0,67 | | -0,04 | NaN | | NaN |  |
| NM_012671 | Tgfa | 9,21 | 9,17 | 9,13 | 9,24 | | 9,28 | 9,34 | 1_0_0 | 0,04 | 0,68 | | 0,04 | 0,66 | | -0,04 |  |
| NM_017124 | Cd37 | 6,50 | 6,46 | 6,49 | 6,76 | | 6,73 | 6,63 | 1_0_0 | 0,00 | 0,68 | | 0,04 | 0,80 | | 0,02 |  |
| NM_053515 | Slc25a4 | 12,87 | 12,89 | 12,82 | 12,90 | | 12,97 | 12,95 | 1_0_0 | 0,05 | 0,68 | | -0,03 | 0,30 | | -0,07 |  |
| NM_024385 | Hhex | 6,67 | 6,70 | 6,88 | 6,86 | | 6,82 | 6,96 | 1_0_0 | 0,01 | 0,69 | | -0,03 | 0,65 | | 0,04 |  |
| NM_138915 | Drd1ip | 8,72 | 8,68 | 8,52 | 8,48 | | 8,49 | 8,45 | 1_0_0 | 0,01 | 0,70 | | 0,04 | 0,96 | | 0,00 |  |
| NM_022301 | Pik4ca | 10,93 | 10,91 | 10,87 | 10,81 | | 10,87 | 10,81 | 1_0_0 | 0,04 | 0,70 | | 0,02 | 0,29 | | -0,06 |  |
| NM_012521 | Calb3 | 6,36 | 6,34 | 6,43 | 6,46 | | 6,44 | 6,53 | 1_0_0 | 0,01 | 0,71 | | 0,02 | 0,78 | | 0,02 |  |
| NM_053621 | Acvrinp1 | 6,83 | 6,86 | 6,86 | 6,95 | | 6,91 | 7,01 | 1_0_0 | 0,05 | 0,71 | | -0,03 | 0,61 | | 0,04 |  |
| NM_031598 | Pla2g2a | 7,64 | 7,45 | 8,05 | 7,05 | | 6,69 | 6,74 | 1_0_0 | 0,01 | 0,73 | | 0,19 | 0,51 | | 0,36 |  |
| NM_139089 | Cxcl10 | 6,92 | 6,80 | 6,76 | 7,36 | | 7,19 | 7,73 | 1_0_0 | 0,01 | 0,74 | | 0,12 | 0,63 | | 0,17 |  |
| NM_213564 | Zbtb9 | 6,88 | 6,90 | 7,01 | 7,04 | | 7,08 | 7,10 | 1_0_0 | 0,01 | 0,75 | | -0,03 | 0,63 | | -0,04 |  |
| NM_024140 | Nrgn | 6,51 | 6,56 | 6,51 | 6,21 | | 6,25 | 6,38 | 1_0_0 | 0,02 | 0,75 | | -0,05 | 0,79 | | -0,04 |  |
| NM_001007725 | Icam2 | 7,41 | 7,44 | 7,38 | 7,62 | | 7,55 | 7,49 | 1_0_0 | 0,02 | 0,76 | | -0,03 | 0,43 | | 0,08 |  |
| NM_031022 | Cspg4 | 8,71 | 8,75 | 8,81 | 8,88 | | 8,94 | 8,99 | 1_0_0 | 0,01 | 0,76 | | -0,03 | 0,55 | | -0,06 |  |
| NM_019186 | Arl4 | 9,97 | 9,99 | 9,97 | 9,87 | | 9,76 | 9,88 | 1_0_0 | 0,01 | 0,77 | | -0,02 | 0,17 | | 0,12 |  |
| NM_031557 | Ptgis | 7,10 | 7,05 | 7,21 | 7,02 | | 6,93 | 6,81 | 1_0_0 | 0,04 | 0,79 | | 0,04 | 0,57 | | 0,09 |  |
| NM_017330 | Prf1 | 6,03 | 6,02 | 6,13 | 6,19 | | 6,11 | 6,11 | 1_0_0 | 0,05 | 0,81 | | 0,02 | NaN | | NaN |  |
| NM_133624 | Gbp2 | 7,91 | 7,87 | 7,86 | 8,14 | | 7,96 | 8,22 | 1_0_0 | 0,02 | 0,81 | | 0,04 | 0,28 | | 0,18 |  |
| NM_001010953 | Rohn | 9,01 | 8,98 | 9,00 | 8,90 | | 8,76 | 8,90 | 1_0_0 | 0,02 | 0,81 | | 0,02 | 0,16 | | 0,14 |  |
| NM_022407 | Aldh1a1 | 10,12 | 10,14 | 10,24 | 10,36 | | 10,23 | 10,54 | 1_0_0 | 0,00 | 0,85 | | -0,02 | 0,26 | | 0,13 |  |
| XM_343268 | Siahbp1 | 11,70 | 11,71 | 11,70 | 11,81 | | 11,73 | 11,79 | 1_0_0 | 0,03 | 0,86 | | -0,01 | 0,14 | | 0,08 |  |
| NM_171992 | Ccnd1 | 9,73 | 9,71 | 9,70 | 9,96 | | 9,85 | 9,84 | 1_0_0 | 0,01 | 0,86 | | 0,02 | 0,31 | | 0,11 |  |
| NM_012591 | Irf1 | 8,72 | 8,71 | 8,74 | 8,83 | | 8,97 | 8,93 | 1_0_0 | 0,00 | 0,87 | | 0,02 | 0,16 | | -0,14 |  |
| NM_012651 | Slc4a1 | 6,16 | 6,17 | 6,17 | 6,06 | | 6,08 | 6,03 | 1_0_0 | 0,02 | 0,87 | | -0,01 | 0,87 | | -0,01 |  |
| XM_576956 | Ace2 | 6,22 | 6,23 | 6,25 | 6,05 | | 6,17 | 6,12 | 1_0_0 | 0,04 | 0,88 | | -0,01 | NaN | | NaN |  |
| NM_053294 | Adora2a | 8,52 | 8,54 | 8,30 | 8,29 | | 8,48 | 8,20 | 1_0_0 | 0,05 | 0,90 | | -0,01 | 0,08 | | -0,19 |  |
| NM_012497 | Aldoc | 12,48 | 12,50 | 12,48 | 12,35 | | 12,33 | 12,39 | 1_0_0 | 0,05 | 0,90 | | -0,01 | 0,80 | | 0,03 |  |
| XM_579085 | Ece2 | 6,30 | 6,29 | 6,26 | 6,34 | | 6,46 | 6,39 | 1_0_0 | 0,04 | 0,91 | | 0,01 | 0,21 | | -0,12 |  |
| NM_013096 | Hba-a1 | 13,91 | 13,90 | 13,93 | 13,29 | | 13,22 | 13,39 | 1_0_0 | 0,00 | 0,92 | | 0,02 | 0,71 | | 0,07 |  |
| NM_012913 | Atp1b3 | 12,89 | 12,89 | 12,93 | 12,95 | | 13,04 | 12,98 | 1_0_0 | 0,02 | 0,93 | | -0,01 | 0,14 | | -0,09 |  |
| NM_145672 | Cxcl9 | 6,93 | 6,96 | 6,75 | 7,57 | | 7,70 | 7,64 | 1_0_0 | 0,01 | 0,94 | | -0,03 | 0,77 | | -0,13 |  |
| NM_032076 | Ptger4 | 6,14 | 6,14 | 6,05 | 6,23 | | 6,24 | 6,16 | 1_0_0 | 0,01 | 0,95 | | 0,00 | 0,85 | | -0,01 |  |
| XM_234422 | c-fos | 6,35 | 6,36 | 6,32 | 6,91 | | 6,60 | 6,61 | 1_0_0 | 0,02 | 0,95 | | -0,01 | 0,21 | | 0,31 |  |
| NM_053356 | Col1a2 | 13,56 | 13,56 | 13,67 | 13,75 | | 13,64 | 13,71 | 1_0_0 | 0,04 | 0,96 | | 0,00 | 0,20 | | 0,10 |  |
| NM_030845 | Cxcl1 | 6,07 | 6,07 | 6,08 | 6,20 | | 6,19 | 6,22 | 1_0_0 | 0,00 | 0,96 | | 0,00 | 0,94 | | 0,00 |  |
| NM_001008315 | Ltbr | 6,34 | 6,34 | 6,37 | 6,42 | | 6,48 | 6,39 | 1_0_0 | 0,04 | 0,96 | | 0,00 | 0,39 | | -0,06 |  |
| NM_012580 | Hmox1 | 7,96 | 7,95 | 7,71 | 8,33 | | 8,09 | 7,83 | 1_0_0 | 0,02 | 0,97 | | 0,01 | 0,10 | | 0,24 |  |
| NM_030868 | Nov | 9,20 | 9,18 | 9,74 | 9,07 | | 8,83 | 8,86 | 1_0_0 | 0,03 | 0,97 | | 0,01 | 0,48 | | 0,23 |  |
| XM_227314 | Sfrp2 | 6,93 | 6,94 | 7,42 | 6,81 | | 6,62 | 6,64 | 1_0_0 | 0,04 | 0,97 | | -0,01 | 0,55 | | 0,19 |  |
| NM_001007148 | Btrc | 7,79 | 7,79 | 7,75 | 7,97 | | 7,87 | 7,82 | 1_0_0 | 0,02 | 0,98 | | 0,00 | 0,21 | | 0,10 |  |
| NM_199382 | Bpgm | 10,89 | 10,89 | 10,82 | 10,64 | | 10,75 | 10,69 | 1_0_0 | 0,00 | 0,98 | | 0,00 | 0,19 | | -0,11 |  |
| NM_177481 | Slco3a1 | 6,59 | 6,59 | 6,55 | 6,67 | | 6,72 | 6,71 | 1_0_0 | 0,03 | 0,98 | | 0,00 | 0,56 | | -0,06 |  |
| XM_342594 | Zbp1 | 6,09 | 6,09 | 5,99 | 6,27 | | 6,34 | 6,25 | 1_0_0 | 0,00 | 0,99 | | 0,00 | 0,54 | | -0,07 |  |
| NM_172017 | Yif1 | 8,99 | 8,99 | 9,02 | 9,09 | | 9,15 | 9,13 | 1_0_0 | 0,01 | 0,99 | | 0,00 | 0,41 | | -0,06 |  |
| NM_012876 | Rps29 | 12,01 | 12,01 | 11,99 | 12,14 | | 12,09 | 12,07 | 1_0_0 | 0,02 | 1,00 | | 0,00 | 0,43 | | 0,06 |  |
| NM_024000 | 1G5 | 6,04 | 5,97 | 6,10 | 5,95 | | 5,92 | 5,99 | 1_0_0 | 0,04 | NaN | | NaN | NaN | | NaN |  |
| NM_031019 | Crh | 7,39 | 7,66 | 7,35 | 7,94 | | 8,10 | 7,84 | 1_0_0 | 0,05 | NaN | | NaN | 0,69 | | -0,16 |  |
| NM_053770 | Argbp2 | 6,71 | 7,32 | 7,12 | 7,48 | | 7,49 | 7,43 | 1_0_0 | 0,01 | NaN | | NaN | NaN | | NaN |  |
| NM_012544 | Ace | 6,05 | 6,11 | 6,04 | 5,97 | | 5,96 | 6,00 | 1_0_0 | 0,04 | NaN | | NaN | NaN | | NaN |  |
| NM_012907 | Apobec1 | 6,07 | 6,07 | 6,02 | 6,19 | | 6,16 | 6,17 | 1_0_0 | 0,01 | NaN | | NaN | NaN | | NaN |  |
| XM_579616 | Ces3 | 6,09 | 6,01 | 6,22 | 6,31 | | 6,26 | 6,33 | 1_0_0 | 0,05 | NaN | | NaN | 0,77 | | 0,04 |  |
| NM_022242 | Niban | 5,91 | 6,09 | 5,94 | 6,08 | | 6,10 | 6,06 | 1_0_0 | 0,01 | NaN | | NaN | 0,67 | | -0,03 |  |
| NM_145670 | Bcas1 | 8,88 | 9,22 | 8,95 | 9,16 | | 8,81 | 9,15 | 0_1_1 | 0,77 | 0,01 | | -0,34 | 0,01 | | 0,35 |  |
| NM_134351 | Mat2a | 6,27 | 6,45 | 6,40 | 6,36 | | 6,19 | 6,39 | 0_1_1 | 0,11 | 0,01 | | -0,18 | 0,01 | | 0,17 |  |
| NM_053854 | Nat2 | 6,04 | 5,92 | 6,03 | 6,01 | | 6,12 | 6,04 | 0_1_1 | 0,05 | 0,02 | | 0,11 | 0,02 | | -0,11 |  |
| NM_053527 | Cdc5l | 9,20 | 9,34 | 9,34 | 9,38 | | 9,18 | 9,32 | 0_1_1 | 0,98 | 0,03 | | -0,14 | 0,00 | | 0,20 |  |
| XM_343564 | Col5a2 | 9,93 | 10,10 | 10,12 | 10,14 | | 9,93 | 10,18 | 0_1_1 | 0,45 | 0,04 | | -0,17 | 0,02 | | 0,21 |  |
| NM_012947 | Eef2k | 8,77 | 7,65 | 8,67 | 7,65 | | 8,92 | 9,18 | 0_1_1 | 0,48 | 0,04 | | 1,12 | 0,03 | | -1,27 |  |
| NM_058208 | Socs2 | 6,33 | 6,13 | 6,10 | 6,12 | | 6,19 | 6,23 | 0_1_0 | 0,85 | 0,00 | | 0,20 | 0,24 | | -0,07 |  |
| NM_001004259 | Pnkp | 6,60 | 6,39 | 6,51 | 6,52 | | 6,55 | 6,46 | 0_1_0 | 0,78 | 0,01 | | 0,21 | 0,63 | | -0,03 |  |
| NM_134398 | P34 | 6,06 | 6,28 | 6,22 | 6,24 | | 6,16 | 6,24 | 0_1_0 | 0,57 | 0,01 | | -0,22 | 0,26 | | 0,08 |  |
| NM_012829 | Cck | 6,26 | 6,53 | 6,29 | 6,34 | | 6,31 | 6,31 | 0_1_0 | 0,48 | 0,01 | | -0,27 | 0,79 | | 0,03 |  |
| **NM_017065** | **Gabrb3** | **5,95** | **6,07** | **6,01** | **6,07** | | **6,05** | **6,06** | **0_1_0** | **0,08** | **0,01** | | **-0,12** | **0,59** | | **0,02** |  |
| NM_001004206 | Pa2g4 | 6,03 | 5,92 | 5,98 | 6,00 | | 5,99 | 5,96 | 0_1_0 | 0,80 | 0,01 | | 0,10 | NaN | | NaN |  |
| NM_017030 | Pccb | 6,70 | 6,50 | 6,60 | 6,66 | | 6,57 | 6,66 | 0_1_0 | 0,48 | 0,01 | | 0,20 | 0,25 | | 0,09 |  |
| NM_001007626 | Ggps1 | 7,43 | 7,18 | 7,26 | 7,20 | | 7,34 | 7,24 | 0_1_0 | 0,61 | 0,02 | | 0,24 | 0,16 | | -0,14 |  |
| NM_053349 | Sox11 | 6,02 | 6,24 | 6,09 | 6,12 | | 6,12 | 6,05 | 0_1_0 | 0,80 | 0,02 | | -0,22 | 0,98 | | 0,00 |  |
| NM_133553 | B3galt4 | 6,42 | 6,26 | 6,42 | 6,41 | | 6,39 | 6,41 | 0_1_0 | 0,30 | 0,02 | | 0,16 | 0,67 | | 0,03 |  |
| NM_203367 | Zmynd11 | 6,20 | 6,40 | 6,48 | 6,43 | | 6,34 | 6,41 | 0_1_0 | 0,50 | 0,02 | | -0,20 | 0,28 | | 0,09 |  |
| NM_001006985 | Mrpl13 | 10,81 | 11,01 | 10,97 | 10,97 | | 10,92 | 10,92 | 0_1_0 | 0,95 | 0,02 | | -0,20 | 0,46 | | 0,06 |  |
| NM_080577 | Nploc4 | 6,09 | 6,24 | 6,18 | 6,18 | | 6,12 | 6,11 | 0_1_0 | 0,35 | 0,03 | | -0,14 | 0,38 | | 0,05 |  |
| NM_013066 | Mtap2 | 6,16 | 6,39 | 6,33 | 6,29 | | 6,18 | 6,35 | 0_1_0 | 0,73 | 0,03 | | -0,22 | 0,27 | | 0,11 |  |
| NM_001007612 | Ccl7 | 6,51 | 6,21 | 6,57 | 6,50 | | 6,51 | 6,45 | 0_1_0 | 0,44 | 0,03 | | 0,30 | 0,91 | | -0,01 |  |
| NM_053719 | Emb | 8,22 | 8,00 | 8,16 | 8,03 | | 8,11 | 8,00 | 0_1_0 | 0,15 | 0,03 | | 0,22 | 0,38 | | -0,09 |  |
| NM_053555 | Vamp5 | 8,05 | 8,25 | 8,18 | 8,15 | | 8,13 | 8,12 | 0_1_0 | 0,62 | 0,03 | | -0,20 | 0,85 | | 0,02 |  |
| NM_053483 | Kpna2 | 9,33 | 9,45 | 9,41 | 9,40 | | 9,49 | 9,41 | 0_1_0 | 0,28 | 0,04 | | -0,12 | 0,10 | | -0,10 |  |
| **NM_080586** | **Gabrg1** | **6,16** | **6,29** | **6,33** | **6,26** | | **6,20** | **6,24** | **0_1_0** | **0,43** | **0,04** | | **-0,13** | **0,31** | | **0,06** |  |
| NM_031345 | Dsipi | 11,74 | 12,02 | 11,78 | 11,86 | | 11,83 | 11,71 | 0_1_0 | 0,52 | 0,04 | | -0,28 | 0,82 | | 0,03 |  |
| NM_012839 | Cycs | 12,27 | 12,44 | 12,40 | 12,33 | | 12,22 | 12,33 | 0_1_0 | 0,11 | 0,04 | | -0,18 | 0,20 | | 0,11 |  |
| NM_001005884 | Letm1 | 8,99 | 9,14 | 9,12 | 9,05 | | 9,15 | 9,04 | 0_1_0 | 1,00 | 0,04 | | -0,15 | 0,15 | | -0,10 |  |
| NM_031129 | Tceb2 | 11,07 | 10,95 | 11,00 | 11,02 | | 11,02 | 10,93 | 0_1_0 | 0,54 | 0,04 | | 0,12 | 0,99 | | 0,00 |  |
| NM_031613 | Tmod2 | 7,74 | 7,94 | 7,82 | 7,81 | | 7,82 | 7,93 | 0_1_0 | 0,68 | 0,04 | | -0,19 | 0,93 | | -0,01 |  |
| **NM_031802** | **Gabbr2** | **11,15** | **11,34** | **11,17** | **11,19** | | **11,19** | **11,22** | **0_1_0** | **0,64** | **0,04** | | **-0,19** | **0,99** | | **0,00** |  |
| NM_030834 | Slc16a3 | 6,94 | 6,66 | 6,76 | 6,73 | | 6,72 | 6,60 | 0_1_0 | 0,19 | 0,04 | | 0,28 | 0,92 | | 0,01 |  |
| NM_053948 | Polr2g | 10,23 | 10,37 | 10,32 | 10,36 | | 10,19 | 10,25 | 0_0_1 | 0,34 | 0,05 | | -0,15 | 0,03 | | 0,17 |  |
| NM_182950 | Tnfaip1 | 6,13 | 6,26 | 6,18 | 6,22 | | 6,02 | 6,20 | 0_0_1 | 0,23 | 0,08 | | -0,13 | 0,01 | | 0,20 |  |
| NM_017182 | H2afy | 10,79 | 10,70 | 10,77 | 10,67 | | 10,81 | 10,73 | 0_0_1 | 0,50 | 0,09 | | 0,09 | 0,02 | | -0,13 |  |
| NM_024145 | Fgr | 6,17 | 6,10 | 6,09 | 6,12 | | 6,02 | 6,10 | 0_0_1 | 0,10 | 0,10 | | 0,07 | 0,02 | | 0,10 |  |
| NM_134402 | Bzw2 | 7,37 | 7,49 | 7,45 | 7,49 | | 7,33 | 7,47 | 0_0_1 | 0,95 | 0,11 | | -0,12 | 0,04 | | 0,16 |  |
| XM_213954 | Nid | 8,22 | 8,94 | 8,75 | 9,44 | | 8,44 | 8,72 | 0_0_1 | 0,38 | 0,12 | | -0,73 | 0,03 | | 1,00 |  |
| XM_231453 | Ophn1 | 6,04 | 6,12 | 5,99 | 6,09 | | 5,97 | 6,00 | 0_0_1 | 0,35 | 0,12 | | -0,08 | 0,03 | | 0,12 |  |
| NM_001006957 | Wbscr1 | 11,90 | 12,01 | 12,04 | 12,02 | | 11,85 | 11,93 | 0_0_1 | 0,18 | 0,12 | | -0,11 | 0,02 | | 0,17 |  |
| NM_019201 | Ctbp1 | 12,55 | 12,46 | 12,49 | 12,42 | | 12,55 | 12,48 | 0_0_1 | 0,63 | 0,13 | | 0,09 | 0,02 | | -0,14 |  |
| NM_054006 | Unr | 7,88 | 7,77 | 7,73 | 7,74 | | 7,94 | 7,75 | 0_0_1 | 0,74 | 0,13 | | 0,11 | 0,01 | | -0,20 |  |
| NM_133290 | Zfp36 | 7,96 | 8,16 | 8,02 | 8,26 | | 7,87 | 8,06 | 0_0_1 | 0,85 | 0,15 | | -0,19 | 0,01 | | 0,39 |  |
| XM_579415 | Slc6a8 | 9,32 | 9,24 | 9,20 | 9,18 | | 9,40 | 9,37 | 0_0_1 | 0,08 | 0,17 | | 0,08 | 0,00 | | -0,22 |  |
| XM_577692 | Dutp | 9,51 | 9,61 | 9,54 | 9,68 | | 9,52 | 9,61 | 0_0_1 | 0,27 | 0,18 | | -0,10 | 0,04 | | 0,16 |  |
| NM_001007146 | Tob2 | 8,44 | 8,61 | 8,60 | 8,61 | | 8,32 | 8,61 | 0_0_1 | 0,63 | 0,18 | | -0,17 | 0,03 | | 0,29 |  |
| NM_012500 | Apeh | 9,22 | 9,14 | 9,17 | 9,08 | | 9,21 | 9,13 | 0_0_1 | 0,28 | 0,21 | | 0,07 | 0,03 | | -0,13 |  |
| NM_178091 | Insig2 | 8,97 | 8,91 | 9,00 | 8,89 | | 9,03 | 8,95 | 0_0_1 | 0,91 | 0,21 | | 0,06 | 0,01 | | -0,14 |  |
| NM_022404 | Gbl | 10,02 | 9,94 | 10,02 | 9,91 | | 10,13 | 9,91 | 0_0_1 | 0,88 | 0,22 | | 0,08 | 0,00 | | -0,22 |  |
| NM_013086 | Crem | 7,16 | 7,23 | 7,15 | 7,23 | | 7,10 | 7,14 | 0_0_1 | 0,52 | 0,22 | | -0,07 | 0,04 | | 0,13 |  |
| NM_024403 | Atf4 | 9,85 | 9,93 | 9,89 | 10,08 | | 9,81 | 9,87 | 0_0_1 | 0,44 | 0,24 | | -0,08 | 0,00 | | 0,26 |  |
| NM_053707 | Hdgf | 10,12 | 10,22 | 10,19 | 10,27 | | 9,96 | 10,18 | 0_0_1 | 0,40 | 0,24 | | -0,10 | 0,00 | | 0,31 |  |
| NM_020308 | Adam15 | 7,51 | 7,38 | 7,36 | 7,32 | | 7,54 | 7,35 | 0_0_1 | 0,82 | 0,26 | | 0,12 | 0,05 | | -0,22 |  |
| NM_053965 | Slc25a20 | 7,87 | 7,75 | 7,77 | 7,79 | | 8,04 | 7,88 | 0_0_1 | 0,09 | 0,26 | | 0,12 | 0,03 | | -0,25 |  |
| NM_031647 | Sfmbt1 | 7,05 | 6,96 | 6,98 | 6,94 | | 7,09 | 7,01 | 0_0_1 | 0,72 | 0,27 | | 0,08 | 0,04 | | -0,16 |  |
| NM_001007682 | Thtpa | 9,35 | 9,42 | 9,38 | 9,46 | | 9,26 | 9,39 | 0_0_1 | 0,66 | 0,27 | | -0,07 | 0,00 | | 0,20 |  |
| NM_199233 | Doxl1 | 6,18 | 6,11 | 6,16 | 6,26 | | 6,10 | 6,13 | 0_0_1 | 0,71 | 0,28 | | 0,06 | 0,01 | | 0,16 |  |
| NM_017294 | Pacsin1 | 7,36 | 7,46 | 7,34 | 7,43 | | 7,20 | 7,37 | 0_0_1 | 0,31 | 0,29 | | -0,10 | 0,03 | | 0,22 |  |
| NM_053341 | Rgs19ip1 | 7,41 | 7,35 | 7,41 | 7,44 | | 7,29 | 7,39 | 0_0_1 | 0,61 | 0,29 | | 0,06 | 0,01 | | 0,15 |  |
| NM_053650 | Pdlim3 | 6,30 | 6,13 | 6,05 | 6,41 | | 6,06 | 6,14 | 0_0_1 | 0,67 | 0,30 | | 0,17 | 0,04 | | 0,35 |  |
| NM_017180 | Phlda1 | 6,42 | 6,30 | 6,40 | 6,55 | | 6,29 | 6,39 | 0_0_1 | 0,57 | 0,31 | | 0,12 | 0,02 | | 0,27 |  |
| NM_019284 | Cspg5 | 8,97 | 8,84 | 8,97 | 9,07 | | 8,80 | 8,91 | 0_0_1 | 1,00 | 0,31 | | 0,13 | 0,05 | | 0,27 |  |
| XM_579385 | G6pdx | 9,44 | 9,52 | 9,46 | 9,61 | | 9,37 | 9,54 | 0_0_1 | 0,41 | 0,31 | | -0,08 | 0,00 | | 0,24 |  |
| XM_340802 | G3bp | 9,09 | 9,17 | 9,19 | 9,22 | | 9,04 | 9,19 | 0_0_1 | 0,92 | 0,32 | | -0,08 | 0,03 | | 0,18 |  |
| XM_342092 | Vars2 | 10,11 | 9,98 | 10,04 | 9,98 | | 10,28 | 10,05 | 0_0_1 | 0,41 | 0,32 | | 0,13 | 0,03 | | -0,30 |  |
| NM_021771 | Trpc3 | 7,79 | 7,71 | 7,76 | 7,61 | | 7,80 | 7,67 | 0_0_1 | 0,17 | 0,32 | | 0,07 | 0,02 | | -0,18 |  |
| NM_199376 | Sil1 | 8,64 | 8,48 | 8,58 | 8,39 | | 8,74 | 8,58 | 0_0_1 | 0,96 | 0,32 | | 0,17 | 0,05 | | -0,34 |  |
| NM_181086 | Tnfrsf12a | 6,94 | 7,02 | 6,91 | 7,17 | | 6,97 | 6,99 | 0_0_1 | 0,09 | 0,32 | | -0,08 | 0,02 | | 0,20 |  |
| XM_579684 | Mrgprf | 6,30 | 6,23 | 6,43 | 6,38 | | 6,18 | 6,39 | 0_0_1 | 0,96 | 0,32 | | 0,08 | 0,02 | | 0,20 |  |
| XM_579181 | Klf2 | 6,75 | 6,85 | 6,88 | 6,94 | | 6,73 | 6,73 | 0_0_1 | 0,60 | 0,32 | | -0,10 | 0,05 | | 0,21 |  |
| NM_013187 | Plcg1 | 9,63 | 9,55 | 9,56 | 9,53 | | 9,76 | 9,55 | 0_0_1 | 0,50 | 0,33 | | 0,08 | 0,01 | | -0,23 |  |
| NM_040669 | Hps1 | 8,11 | 8,19 | 8,17 | 8,03 | | 8,23 | 8,13 | 0_0_1 | 0,55 | 0,35 | | -0,07 | 0,02 | | -0,20 |  |
| NM_134410 | Mg87 | 9,59 | 9,65 | 9,71 | 9,68 | | 9,50 | 9,67 | 0_0_1 | 0,38 | 0,35 | | -0,06 | 0,01 | | 0,19 |  |
| XM_343117 | Siva | 8,43 | 8,48 | 8,46 | 8,62 | | 8,40 | 8,52 | 0_0_1 | 0,12 | 0,38 | | -0,06 | 0,00 | | 0,21 |  |
| NM_022631 | Wnt5a | 6,29 | 6,21 | 6,30 | 6,34 | | 6,15 | 6,27 | 0_0_1 | 0,79 | 0,39 | | 0,07 | 0,03 | | 0,19 |  |
| NM_199495 | Ndufa10 | 12,12 | 11,99 | 12,03 | 11,84 | | 12,22 | 11,97 | 0_0_1 | 0,68 | 0,39 | | 0,13 | 0,02 | | -0,39 |  |
| NM_001008520 | Abhd1 | 8,78 | 8,70 | 8,61 | 8,69 | | 8,92 | 8,71 | 0_0_1 | 0,17 | 0,40 | | 0,08 | 0,03 | | -0,23 |  |
| NM_134349 | Mgst1 | 10,78 | 10,71 | 10,70 | 10,82 | | 10,64 | 10,78 | 0_0_1 | 0,75 | 0,40 | | 0,07 | 0,04 | | 0,18 |  |
| NM_199384 | Laptm4a | 13,04 | 13,08 | 13,13 | 13,13 | | 13,00 | 13,13 | 0_0_1 | 0,91 | 0,40 | | -0,04 | 0,01 | | 0,13 |  |
| NM_017279 | Psma2 | 11,55 | 11,61 | 11,62 | 11,63 | | 11,42 | 11,56 | 0_0_1 | 0,16 | 0,41 | | -0,05 | 0,00 | | 0,21 |  |
| NM_031050 | Lum | 11,31 | 11,42 | 11,54 | 11,53 | | 11,23 | 11,50 | 0_0_1 | 0,98 | 0,41 | | -0,11 | 0,03 | | 0,30 |  |
| NM_032067 | Ralbp1 | 10,72 | 10,77 | 10,74 | 10,67 | | 10,86 | 10,81 | 0_0_1 | 0,33 | 0,42 | | -0,05 | 0,01 | | -0,19 |  |
| XM_579721 | Prg-2 | 6,28 | 6,22 | 6,21 | 6,14 | | 6,38 | 6,26 | 0_0_1 | 0,62 | 0,43 | | 0,06 | 0,00 | | -0,25 |  |
| NM_012843 | Emp1 | 9,09 | 9,19 | 9,42 | 9,37 | | 9,09 | 9,31 | 0_0_1 | 0,76 | 0,45 | | -0,10 | 0,04 | | 0,28 |  |
| NM_053667 | Lepre1 | 8,95 | 8,90 | 8,88 | 8,89 | | 9,03 | 8,90 | 0_0_1 | 0,42 | 0,46 | | 0,05 | 0,04 | | -0,14 |  |
| NM_148891 | Nmt1 | 8,73 | 8,69 | 8,68 | 8,79 | | 8,64 | 8,65 | 0_0_1 | 0,85 | 0,47 | | 0,04 | 0,02 | | 0,14 |  |
| NM_080478 | Apbb1 | 7,86 | 7,78 | 7,75 | 7,76 | | 8,00 | 7,82 | 0_0_1 | 0,31 | 0,50 | | 0,07 | 0,04 | | -0,24 |  |
| NM_199108 | Hp1bp3 | 11,60 | 11,63 | 11,70 | 11,58 | | 11,71 | 11,69 | 0_0_1 | 0,40 | 0,50 | | -0,03 | 0,01 | | -0,13 |  |
| NM_144561 | Fmo4 | 6,82 | 6,88 | 6,82 | 6,72 | | 6,90 | 6,82 | 0_0_1 | 0,54 | 0,50 | | -0,06 | 0,04 | | -0,18 |  |
| NM_001005560 | Pla2g6 | 8,85 | 8,89 | 8,81 | 8,96 | | 8,83 | 8,83 | 0_0_1 | 0,41 | 0,50 | | -0,04 | 0,03 | | 0,12 |  |
| NM_053506 | Hrh3 | 6,20 | 6,24 | 6,28 | 6,21 | | 6,37 | 6,18 | 0_0_1 | 0,69 | 0,50 | | -0,04 | 0,01 | | -0,17 |  |
| NM_022260 | Casp7 | 7,75 | 7,81 | 7,80 | 7,99 | | 7,79 | 7,82 | 0_0_1 | 0,14 | 0,51 | | -0,06 | 0,04 | | 0,20 |  |
| NM_031097 | Rnpep | 9,73 | 9,69 | 9,76 | 9,71 | | 9,87 | 9,76 | 0_0_1 | 0,17 | 0,52 | | 0,04 | 0,03 | | -0,16 |  |
| XM_579615 | Hadha | 9,60 | 9,55 | 9,54 | 9,50 | | 9,70 | 9,52 | 0_0_1 | 0,84 | 0,53 | | 0,06 | 0,03 | | -0,20 |  |
| NM_001009661 | Wbp11 | 8,69 | 8,73 | 8,75 | 8,81 | | 8,63 | 8,71 | 0_0_1 | 0,84 | 0,54 | | -0,05 | 0,02 | | 0,18 |  |
| NM_001007147 | Unc84a | 6,22 | 6,25 | 6,28 | 6,30 | | 6,18 | 6,21 | 0_0_1 | 0,49 | 0,54 | | -0,03 | 0,04 | | 0,12 |  |
| XM_579426 | Dnch1 | 13,43 | 13,40 | 13,39 | 13,39 | | 13,54 | 13,38 | 0_0_1 | 0,42 | 0,56 | | 0,03 | 0,01 | | -0,15 |  |
| NM_138850 | Fap | 6,65 | 6,70 | 6,86 | 6,87 | | 6,61 | 6,85 | 0_0_1 | 0,42 | 0,57 | | -0,05 | 0,01 | | 0,26 |  |
| NM_130431 | Hspb2 | 7,63 | 7,58 | 7,59 | 7,74 | | 7,53 | 7,62 | 0_0_1 | 0,58 | 0,58 | | 0,05 | 0,02 | | 0,21 |  |
| NM_057143 | Park7 | 12,18 | 12,15 | 12,17 | 12,11 | | 12,22 | 12,15 | 0_0_1 | 0,82 | 0,59 | | 0,03 | 0,04 | | -0,11 |  |
| XM_235707 | Itga5 | 6,54 | 6,50 | 6,46 | 6,63 | | 6,42 | 6,48 | 0_0_1 | 0,83 | 0,62 | | 0,04 | 0,03 | | 0,20 |  |
| NM_080397 | Chst10 | 8,40 | 8,43 | 8,43 | 8,38 | | 8,56 | 8,41 | 0_0_1 | 0,54 | 0,62 | | -0,03 | 0,01 | | -0,18 |  |
| NM_144758 | Slc15a4 | 9,53 | 9,50 | 9,37 | 9,44 | | 9,58 | 9,41 | 0_0_1 | 0,80 | 0,63 | | 0,03 | 0,03 | | -0,14 |  |
| XM_223843 | Rarb | 6,37 | 6,33 | 6,32 | 6,41 | | 6,21 | 6,37 | 0_0_1 | 0,86 | 0,63 | | 0,04 | 0,02 | | 0,20 |  |
| NM_031154 | Gstm3 | 9,70 | 9,75 | 9,80 | 9,83 | | 9,60 | 9,75 | 0_0_1 | 0,62 | 0,64 | | -0,05 | 0,04 | | 0,23 |  |
| NM_012984 | Myo9b | 9,45 | 9,40 | 9,33 | 9,25 | | 9,56 | 9,36 | 0_0_1 | 0,97 | 0,65 | | 0,05 | 0,01 | | -0,31 |  |
| NM_134369 | Cyp2t1 | 6,92 | 6,96 | 6,94 | 6,82 | | 7,05 | 6,99 | 0_0_1 | 0,81 | 0,65 | | -0,04 | 0,03 | | -0,23 |  |
| NM_012851 | Hsd17b1 | 6,15 | 6,18 | 6,12 | 6,27 | | 6,13 | 6,21 | 0_0_1 | 0,19 | 0,65 | | -0,03 | 0,05 | | 0,13 |  |
| NM_181432 | Hps6 | 9,20 | 9,16 | 9,23 | 9,18 | | 9,37 | 9,20 | 0_0_1 | 0,24 | 0,65 | | 0,04 | 0,03 | | -0,19 |  |
| NM_031094 | Rbl2 | 8,48 | 8,43 | 8,51 | 8,36 | | 8,64 | 8,54 | 0_0_1 | 0,56 | 0,65 | | 0,05 | 0,02 | | -0,28 |  |
| NM_139333 | Prp19 | 9,45 | 9,40 | 9,45 | 9,49 | | 9,26 | 9,36 | 0_0_1 | 0,33 | 0,66 | | 0,05 | 0,04 | | 0,23 |  |
| NM_022244 | Arhgap17 | 10,53 | 10,51 | 10,59 | 10,51 | | 10,63 | 10,57 | 0_0_1 | 0,28 | 0,67 | | 0,02 | 0,03 | | -0,12 |  |
| NM_017336 | Ptpro | 9,25 | 9,21 | 9,17 | 9,03 | | 9,30 | 9,10 | 0_0_1 | 0,20 | 0,67 | | 0,04 | 0,01 | | -0,27 |  |
| NM_183325 | Adprh | 9,66 | 9,68 | 9,69 | 9,73 | | 9,59 | 9,68 | 0_0_1 | 0,77 | 0,68 | | -0,02 | 0,02 | | 0,13 |  |
| NM_012945 | Dtr | 7,82 | 7,86 | 7,74 | 7,99 | | 7,77 | 7,79 | 0_0_1 | 0,38 | 0,69 | | -0,03 | 0,02 | | 0,21 |  |
| NM_019248 | Ntrk3 | 9,62 | 9,66 | 9,66 | 9,49 | | 9,70 | 9,58 | 0_0_1 | 0,30 | 0,70 | | -0,03 | 0,03 | | -0,21 |  |
| NM_031698 | Rpn2 | 10,08 | 10,04 | 10,00 | 10,16 | | 9,94 | 10,03 | 0_0_1 | 0,91 | 0,71 | | 0,04 | 0,04 | | 0,22 |  |
| NM_134452 | Col5a1 | 9,44 | 9,47 | 9,50 | 9,63 | | 9,41 | 9,54 | 0_0_1 | 0,28 | 0,73 | | -0,03 | 0,03 | | 0,22 |  |
| NM_022691 | Exoc7 | 8,33 | 8,35 | 8,38 | 8,27 | | 8,41 | 8,40 | 0_0_1 | 0,83 | 0,73 | | -0,02 | 0,02 | | -0,15 |  |
| NM_138911 | Stip1 | 9,46 | 9,43 | 9,30 | 9,63 | | 9,38 | 9,29 | 0_0_1 | 0,50 | 0,73 | | 0,03 | 0,01 | | 0,25 |  |
| NM_173154 | Asam | 7,94 | 7,91 | 7,92 | 7,80 | | 8,03 | 7,84 | 0_0_1 | 0,60 | 0,74 | | 0,03 | 0,03 | | -0,23 |  |
| NM_001004089 | Sipa1 | 6,58 | 6,55 | 6,45 | 6,65 | | 6,47 | 6,52 | 0_0_1 | 0,72 | 0,74 | | 0,03 | 0,04 | | 0,18 |  |
| NM_024155 | Anxa4 | 11,28 | 11,31 | 11,32 | 11,40 | | 11,16 | 11,32 | 0_0_1 | 0,85 | 0,75 | | -0,03 | 0,02 | | 0,23 |  |
| XM_217297 | Acvr2b | 8,56 | 8,53 | 8,50 | 8,38 | | 8,57 | 8,49 | 0_0_1 | 0,36 | 0,76 | | 0,03 | 0,04 | | -0,19 |  |
| NM_173126 | Nid67 | 8,91 | 8,93 | 8,97 | 9,07 | | 8,92 | 8,97 | 0_0_1 | 0,19 | 0,76 | | -0,02 | 0,03 | | 0,15 |  |
| NM_133283 | Map2k2 | 11,69 | 11,67 | 11,68 | 11,63 | | 11,82 | 11,69 | 0_0_1 | 0,48 | 0,76 | | 0,02 | 0,04 | | -0,18 |  |
| XM_579648 | Ero1l | 6,36 | 6,39 | 6,43 | 6,51 | | 6,23 | 6,35 | 0_0_1 | 0,69 | 0,78 | | -0,04 | 0,04 | | 0,28 |  |
| NM_053395 | Smpx | 6,91 | 6,93 | 6,96 | 6,87 | | 7,04 | 6,86 | 0_0_1 | 0,77 | 0,78 | | -0,02 | 0,01 | | -0,16 |  |
| NM_013180 | Itgb4 | 10,53 | 10,49 | 10,64 | 10,43 | | 10,70 | 10,61 | 0_0_1 | 0,70 | 0,78 | | 0,03 | 0,04 | | -0,27 |  |
| NM_022526 | Dap | 8,97 | 8,95 | 9,13 | 9,09 | | 8,91 | 9,06 | 0_0_1 | 0,84 | 0,79 | | 0,02 | 0,01 | | 0,18 |  |
| NM_199117 | Cbx7 | 9,09 | 9,07 | 9,11 | 8,98 | | 9,21 | 9,14 | 0_0_1 | 0,62 | 0,80 | | 0,02 | 0,01 | | -0,24 |  |
| NM_198726 | Kpna1 | 9,65 | 9,69 | 9,67 | 9,61 | | 9,91 | 9,61 | 0_0_1 | 0,60 | 0,80 | | -0,03 | 0,04 | | -0,29 |  |
| NM_001005761 | Mtmr9 | 7,80 | 7,82 | 7,87 | 7,98 | | 7,76 | 7,88 | 0_0_1 | 0,38 | 0,80 | | -0,02 | 0,02 | | 0,22 |  |
| NM_139111 | Cklf1 | 7,34 | 7,31 | 7,44 | 7,47 | | 7,17 | 7,32 | 0_0_1 | 0,54 | 0,80 | | 0,03 | 0,02 | | 0,30 |  |
| NM_012792 | Fmo1 | 8,50 | 8,52 | 8,60 | 8,34 | | 8,64 | 8,48 | 0_0_1 | 0,43 | 0,81 | | -0,03 | 0,01 | | -0,30 |  |
| NM_212490 | Atp6v1g2 | 12,08 | 12,07 | 12,12 | 12,02 | | 12,18 | 12,01 | 0_0_1 | 0,59 | 0,81 | | 0,02 | 0,04 | | -0,16 |  |
| NM_134417 | Impk | 7,93 | 7,94 | 7,96 | 7,85 | | 8,00 | 8,00 | 0_0_1 | 0,80 | 0,82 | | -0,01 | 0,01 | | -0,15 |  |
| NM_053867 | Tpt1 | 13,76 | 13,78 | 13,80 | 13,86 | | 13,66 | 13,78 | 0_0_1 | 0,85 | 0,82 | | -0,02 | 0,04 | | 0,20 |  |
| NM_053713 | Klf4 | 7,50 | 7,54 | 7,68 | 7,76 | | 7,40 | 7,49 | 0_0_1 | 0,82 | 0,83 | | -0,04 | 0,04 | | 0,36 |  |
| NM_139080 | G22p1 | 8,95 | 8,97 | 8,99 | 9,12 | | 8,92 | 8,98 | 0_0_1 | 0,45 | 0,84 | | -0,02 | 0,04 | | 0,20 |  |
| NM_001008281 | Psmd3 | 11,40 | 11,38 | 11,43 | 11,25 | | 11,53 | 11,35 | 0_0_1 | 0,75 | 0,85 | | 0,03 | 0,04 | | -0,28 |  |
| NM_022219 | Fut4 | 6,68 | 6,66 | 6,72 | 6,60 | | 6,80 | 6,75 | 0_0_1 | 0,49 | 0,85 | | 0,01 | 0,02 | | -0,20 |  |
| NM_031792 | Spag4 | 6,90 | 6,89 | 6,96 | 6,81 | | 6,98 | 6,80 | 0_0_1 | 0,25 | 0,85 | | 0,01 | 0,04 | | -0,17 |  |
| NM_021863 | Hspa2 | 9,63 | 9,65 | 9,56 | 9,80 | | 9,51 | 9,75 | 0_0_1 | 0,28 | 0,86 | | -0,02 | 0,02 | | 0,29 |  |
| NM_178106 | Entpd3 | 6,89 | 6,91 | 6,90 | 7,03 | | 6,86 | 6,93 | 0_0_1 | 0,29 | 0,86 | | -0,01 | 0,02 | | 0,17 |  |
| NM_013044 | Tmod1 | 11,01 | 10,99 | 10,91 | 10,84 | | 11,08 | 10,92 | 0_0_1 | 0,68 | 0,88 | | 0,01 | 0,03 | | -0,23 |  |
| NM_031708 | Adrm1 | 7,79 | 7,81 | 7,78 | 7,97 | | 7,69 | 7,79 | 0_0_1 | 0,66 | 0,89 | | -0,01 | 0,01 | | 0,28 |  |
| NM_133577 | Gpr105 | 6,87 | 6,89 | 6,98 | 7,00 | | 6,81 | 7,00 | 0_0_1 | 0,64 | 0,89 | | -0,01 | 0,04 | | 0,19 |  |
| NM_177929 | Sdccag8 | 6,99 | 6,98 | 6,97 | 6,95 | | 7,12 | 7,02 | 0_0_1 | 0,24 | 0,90 | | 0,01 | 0,02 | | -0,17 |  |
| NM_031721 | Prss11 | 12,34 | 12,35 | 12,28 | 12,30 | | 12,45 | 12,33 | 0_0_1 | 0,38 | 0,90 | | -0,01 | 0,05 | | -0,15 |  |
| NM_022674 | H2afz | 11,28 | 11,29 | 11,26 | 11,33 | | 11,16 | 11,27 | 0_0_1 | 0,65 | 0,91 | | -0,01 | 0,04 | | 0,16 |  |
| NM_001009268 | Actr2 | 12,82 | 12,82 | 12,84 | 12,85 | | 12,71 | 12,81 | 0_0_1 | 0,31 | 0,91 | | 0,01 | 0,05 | | 0,14 |  |
| NM_031024 | Dbn1 | 6,54 | 6,55 | 6,50 | 6,49 | | 6,69 | 6,51 | 0_0_1 | 0,30 | 0,92 | | -0,01 | 0,00 | | -0,19 |  |
| NM_053500 | Slc25a27 | 8,16 | 8,15 | 8,13 | 8,05 | | 8,21 | 8,13 | 0_0_1 | 0,71 | 0,95 | | 0,00 | 0,04 | | -0,15 |  |
| NM_001007686 | Cln8 | 8,76 | 8,75 | 8,69 | 8,69 | | 8,84 | 8,77 | 0_0_1 | 0,45 | 0,95 | | 0,00 | 0,04 | | -0,15 |  |
| NM_031978 | Psmd1 | 10,58 | 10,58 | 10,61 | 10,64 | | 10,50 | 10,54 | 0_0_1 | 0,42 | 0,96 | | 0,00 | 0,02 | | 0,14 |  |
| NM_001006962 | Tinf2 | 7,25 | 7,25 | 7,25 | 7,23 | | 7,36 | 7,36 | 0_0_1 | 0,08 | 0,96 | | 0,00 | 0,04 | | -0,13 |  |
| NM_017192 | Edg5 | 8,26 | 8,25 | 8,33 | 8,45 | | 8,19 | 8,34 | 0_0_1 | 0,41 | 0,97 | | 0,00 | 0,02 | | 0,25 |  |
| NM_001004443 | Hexa | 9,50 | 9,50 | 9,53 | 9,66 | | 9,48 | 9,60 | 0_0_1 | 0,15 | 0,98 | | 0,00 | 0,03 | | 0,18 |  |
| NM_017035 | Plcd1 | 9,01 | 9,01 | 8,88 | 8,87 | | 9,04 | 8,91 | 0_0_1 | 0,49 | 0,99 | | 0,00 | 0,03 | | -0,17 |  |
| NM_181478 | Rdh10 | 6,06 | 6,08 | 6,08 | 6,10 | | 5,94 | 6,05 | 0_0_1 | 0,20 | NaN | | NaN | 0,01 | | 0,16 |  |
